# Quinol derivatives as potential trypanocidal agents

**DOI:** 10.1016/j.bmc.2011.12.018

**Published:** 2012-02-15

**Authors:** Amy Capes, Stephen Patterson, Susan Wyllie, Irene Hallyburton, Iain T. Collie, Andrew J. McCarroll, Malcolm F.G. Stevens, Julie A. Frearson, Paul G. Wyatt, Alan H. Fairlamb, Ian H. Gilbert

**Affiliations:** a Division of Biological Chemistry and Drug Discovery, College of Life Sciences, University of Dundee, Dundee DD1 5EH, UK; b Pharminox Ltd, Biocity, Pennyfoot St., Nottingham NG1 1GF, UK; c Centre for Biomolecular Sciences, School of Pharmacy, University of Nottingham, Nottingham NG7 2RD, UK

**Keywords:** Inhibitors, Medicinal chemistry, *Trypanosoma brucei*, P2 transporter, Quinols

## Abstract

Quinols have been developed as a class of potential anti-cancer compounds. They are thought to act as double Michael acceptors, forming two covalent bonds to their target protein(s). Quinols have also been shown to have activity against the parasite *Trypanosoma brucei*, the causative organism of human African trypanosomiasis, but they demonstrated little selectivity over mammalian MRC5 cells in a counter-screen. In this paper, we report screening of further examples of quinols against *T. brucei*. We were able to derive an SAR, but the compounds demonstrated little selectivity over MRC5 cells. In an approach to increase selectivity, we attached melamine and benzamidine motifs to the quinols, because these moieties are known to be selectively concentrated in the parasite by transporter proteins. In general these transporter motif-containing analogues showed increased selectivity; however they also showed reduced levels of potency against *T. brucei*.

## Graphical abstract

**Figure:**
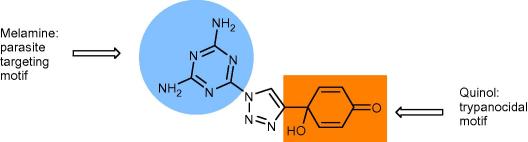

## 1 Introduction

Human African trypanosomiasis (HAT) or sleeping sickness, caused by the parasite *Trypanosoma brucei*, is a major health burden in sub-Saharan Africa.^1^ Currently, there are four licensed drugs^2^ and one licensed drug combination^3^ for the treatment of HAT, all of which suffer from problems such as high toxicity, inappropriate administration in a rural African setting, or the emergence of drug resistance. HAT is invariably fatal if left untreated, therefore the need for new drugs is urgent.^1^

Quinols (compounds containing a 4-hydroxycyclohexa-2,5-dien-1-one moiety) have been investigated as potential anti-cancer agents.^4^, ^5^ The quinol pharmacophore was discovered during investigation of polyphenol tyrphostin kinase inhibitors; the polyphenols were unstable with respect to oxidation and it was noticed that the resultant quinol oxidation products were more potent than the parent compounds.^6^ Although work has continued on the ‘parent’ phenolic compounds,^7^, ^8^, ^9^, ^10^ the quinol pharmacophore was identified as the active moiety, and a series of compounds bearing this novel motif were found to be active against human colon and breast cancer cell lines.^11^, ^12^

**Scheme 1:**
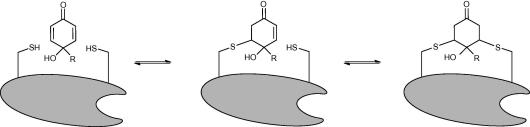
Proposed mode of action of the quinols. Cysteine residues are abbreviated to -SH.

The quinol moiety is a double Michael acceptor, which is able to react with nucleophiles in various cellular proteins, and is thought to bind preferentially to vicinal cysteines (Scheme 1).^5^, ^13^, ^14^ Quinol protein targets have been shown to include β-tubulin, heat shock protein 60, peroxiredoxin 1,^15^ *Mycobacterium tuberculosis* thioredoxin C^16^ and the redox-regulatory protein thioredoxin (Trx).^5^, ^13^ Thioredoxin reductase (TrxR) has also been identified as a target, although in the case of TrxR the quinol motif forms a bond with a nucleophilic selenocysteine residue in the active site.^11^, ^12^ The thioredoxin system is important in countering oxidative stress in the cell, and inhibition of its function eventually leads to apoptosis.^13^, ^17^

While *T. brucei* does not have a thioredoxin reductase/thioredoxin system it possesses an analogous thiol metabolism that is only found in trypanosomatids, including *Trypanosoma* and *Leishmania* species.^18^ The active metabolite of the drug melarsoprol, melarsen oxide, is also known to bind irreversibly to vicinal cysteines such as those in trypanothione^19^ as well as to other substrates containing a similar arrangement of cysteines.^20^ Because melarsoprol is a very potent trypanocide, compounds containing a quinol motif may also show trypanocidal activity. We have previously reported data for seven quinol analogues against *T. brucei*, and established that these compounds form stable complexes with the anti-oxidant trypanothione, as well as inhibiting two classes of tryparedoxin peroxidase.^21^ In this paper, we report the screening of a larger library of quinols to establish a more comprehensive SAR. These compounds showed anti-parasitic activity, but no selectivity compared to human MRC5 cells. We then describe our approaches to achieve selectivity.

## 2 Results and discussion

### 2.1 Screening

We report data for an additional 17 quinol analogues screened against blood stream form *T. brucei*. This quinol collection represents a subset of known compounds^22^, ^23^ in addition to two new analogues (**3** and **12**). The synthesis of the latter two molecules is described below. These latter compounds were prepared to further investigate the SAR; their synthesis is shown in the chemistry section. Together with our previously reported assay data,^21^ this allows us to derive some SAR trends over several sub-series of quinols. The results of this screening are shown in Table 1.

In general, the quinols showed good activity against *T. brucei*, with several EC_50_ values in the low nanomolar range (**6**, **10**, **15**, **16**, **18**, **20**–**25**), comparable in potency to pentamidine (8 nM) and melarsoprol (33 nM) assayed under identical conditions. However it is also apparent that there was essentially no selectivity over the human MRC5 cells, with most compounds showing a ratio of EC_50_ (MRC5)/EC_50_ (*T. brucei*) of ⩽3. The only compounds showing a marginally higher selectivity were also the least active analogues (**11**, **12**). This poor selectivity ratio could be because the compounds have so far been optimised for their activity against human cancer cell lines. Whilst these compounds potentially have value in targeting cancer cells, their selectivity is not sufficient for treating trypanosomiasis, based on our cellular studies. Therefore, a method for conferring selectivity on the compounds is essential if they are to be considered as potential trypanocides.

A few generalisations can be made from this data, which mirrors what has been reported in cancer cell models:

• The quinol moiety is essential for activity. Compare the phenol **7** which is inactive, with its quinol homologue **4** (PMX 464), which showed an EC_50_ of 77 nM against *T. brucei*.^21^
• There is some room for variation around the quinol ring, as has been reported against cancer cell lines^12^: for example replacing the hydroxyl group with a methoxy group only gave a small (fourfold) change in potency (compare **4** and **5**); addition of a chlorine atom to the quinol moiety had no effect on activity (compare **4** and **6**).
• The nature of the aromatic substituent on the quinol is important for potency, but not selectivity. Replacing a phenyl (**2**) with a 2-benzothiazole (**4**) led to a 10-fold increase in potency. Compounds with a benzothiazole group (**4**–**6**; 46–330 nM range) or an indolyl-sulfonamide linked to the quinol (**13**–**25**, 12–170 nM range) were generally potent, confirming the previous observation that the indolyl enhances the potency of these compounds.^25^, ^26^ The compounds containing a triazole linker with different groups at the 1-position (**8**–**12**) have a range of potencies over three orders of magnitude. This again broadly reflects the results against cancer cell lines.^23^

### 2.2 Selectively targeting quinols to *T. brucei*

One possible strategy to circumvent the poor selectivity of the quinols for *T. brucei* over MRC5 cells is to selectively target them to the trypanosome. We decided to investigate this strategy by attaching melamine and benzamidine moieties to the quinols. *T. brucei* is auxotrophic for all purines, which it scavenges from the bloodstream of its host. In order to do this, *T. brucei* has a range of nucleobase transporters in the cell membrane.^27^ The first such transporter characterised was the P2 transporter.^28^ In addition to the uptake of the physiological substrates adenine and adenosine, the P2 transporter is also able to take up compounds containing melamine and benzamidine moieties. Thus it is involved in the selective concentration of the drugs melarsoprol, pentamidine and berenil into trypanosomes. Melarsoprol and pentamidine are used for treating HAT and berenil is a treatment for animal trypanosomiasis. As the biology has been further investigated other transporters involved in the uptake of melamine and benzamidine moieties in trypanosomes have been discovered, such as HAPT1 and LAPT1.^29^ Nevertheless, melamine and benzamidine moieties are selectively taken up into trypanosomes and this strategy has been used to selectively target compounds to trypanosomes, with considerable success in some cases.^30^

The SAR studies above were used to inform where the benzamidine or melamine targeting motif should be attached to the pharmacophore. Attachment of substituents via an acetylene linker did not give potent compounds (**1** and **3**), and modification of the benzothiazole appeared problematic. Attaching the targeting motif to the triazole would be feasible and result in relatively small molecules; however, it would need to be attached directly to the triazole, rather than through a linker (compare **8** and **9** with **12**). Similarly, attachment of the P2 motif to the R_1_ position of the indolyl would also be synthetically feasible, although it would produce larger molecules.

### 2.3 Chemistry

#### 2.3.1 Synthesis of quinol analogues

**Scheme 2:**
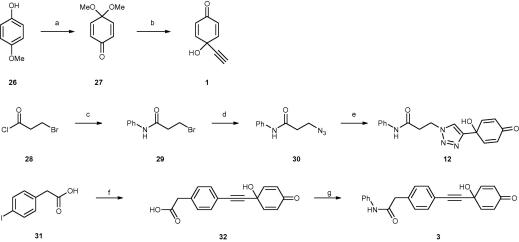
Preparation of additional quinol analogues. Reagents and conditions: (a) PIDA, MeOH, 76%; (b) (i) HC≡CMgBr, THF, −78 °C; (ii) CHCl_3_, silica, 90%; (c) aniline, pyridine, CH_2_Cl_2_, 0–25 °C, 1 h, 69%; (d) NaN_3_, DMF, 60 °C, 16 h, 66%; (e) **1**, CuI, lutidine, MeCN, 0–25 °C, 16 h, 54%; (f) **1**, CuI, Pd(PPh_3_)_4_, DMAc, H_2_O, *i*Pr_2_NH, 100 °C, 10 min, 11%; (g) aniline, PyBrop, DMF, DIPEA, 0–25 °C, 16 h, 20%.

Analogues **3** and **12** were synthesised as outlined in Scheme 2. Compounds **3** and **12** were designed to investigate additional positions at which a benzamidine or melamine moiety could be introduced into the quinol pharmacophore. The 4-ethynyl substituted quinol **1** was prepared as previously described in the literature^31^ (Scheme 2).

The triazole-containing quinol **12** was prepared by the use of a copper catalysed azide alkyne Huisgen cycloaddition reaction (click chemistry)^32^ between azide **30** and alkyne **1**. Azide **30** was prepared from acid chloride **28** following a literature route.^33^ Analogue **3** contains an alkyne linker, which was introduced in the first step of the synthesis using a Sonogashira coupling reaction. Aryl iodide **31** was coupled with alkyne **1** and the resultant acid **32** was converted to the amide **3** by reaction with aniline and PyBrop.

#### 2.3.2 Synthesis of P2 transporter motif-containing quinol analogues

**Scheme 3:**
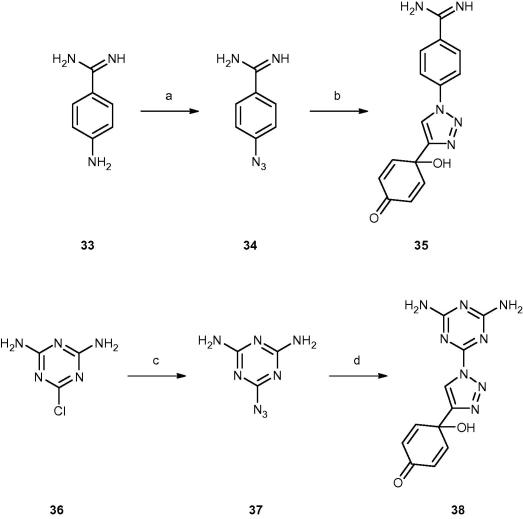
Preparation of triazole quinol analogues containing a benzamidine or a melamine moiety. Reagents and conditions: (a) NaNO_2_, NaN_3_, H_2_SO_4_, H_2_O, 0 °C, 56%; (b) **1**, CuI, lutidine, MeCN, 4%; (c) NaN_3_, Et_3_N, DMF/H_2_O, 115 °C, 31%; (d) **1**, CuI, lutidine, MeCN, 3%.

**Scheme 4:**
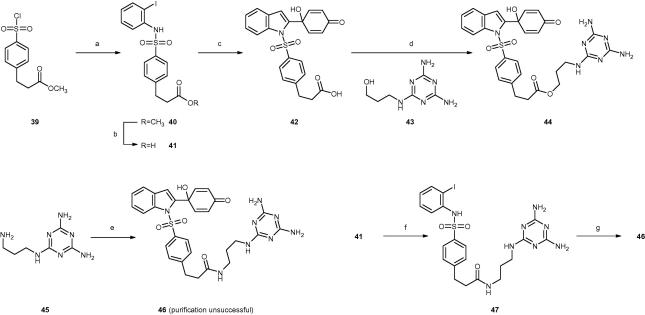
Preparation of indolyl quinol analogues containing a melamine moiety. Reagents and conditions: (a) 2-Iodoaniline, pyridine, THF, 25 °C, 16 h, 62%; (b) (i) KOH, H_2_O, 100 °C, 1 h; (ii) HCl, H_2_O, 0 °C, 1 h, 95%; (c) **1**, CuI, Pd(PPh_3_)_4_, DMAc, H_2_O, *^i^*Pr_2_NH, 100 °C, 10 min, 14%; (d) DIC, DIPEA, DMF, 0–25 °C, 18 h, 67%; (e) **42**, TBTU, HOAt, DIPEA, DMF, 25 °C, 16 h, purification not successful; (f) **45**, TBTU, HOAt, DIPEA, DMF, 25 °C, 16 h, 11%; (g) **1**, CuI, Pd(PPh_3_)_4_, DMF, H_2_O, *^i^*Pr_2_NH, 100 °C (MW), 10 min, 40%.

Two strategies were investigated for attaching the quinol pharmacophore to the melamine and benzamidine P2 targeting motifs. In the first approach (Scheme 3), the melamine and benzamidine moieties were attached via a triazole ring using click chemistry. In the second approach, the targeting moiety was introduced as a substituent to the indolylsulphonamides (Scheme 4), because these were amongst the most potent derivatives. In addition, the SAR indicates a broad tolerance to substituents at the *para*-position of the phenylsulfonyl ring (e.g. **16**–**25**).

Compounds **35** and **38** were synthesised using a copper catalysed click reaction for the final step. The requisite azido benzamidine (**34**) was prepared by diazotization and subsequent substitution of **33** with sodium azide,^34^ while the triazine azide was accessed via an S_N_AR of the chloro-diamino-triazine **36** with sodium azide.^35^ These were then reacted with the quinol alkyne **1**.^31^ The click chemistry reactions were carried out using lutedine and CuI under anhydrous conditions to prevent N_2_ loss,^36^ which was found to be a problem when using standard reaction conditions (CuSO_4_, sodium ascorbate, water/MeCN).

The indolyl-sulphonamide scaffold was prepared as shown in Scheme 4, starting from the commercially available sulfonyl chloride **39** using a modification of a previously reported methodology.^22^ A carboxylic acid was used as a ‘handle’ to attach the melamine moiety by use of amide/ester coupling reactions. However, the previously reported syntheses of acid **42** involved a poor yielding trimethyltin hydroxide mediated hydrolysis of the corresponding methyl ester (**19**).^25^ Therefore, we attempted the Sonogashira coupling-indole forming cascade between the known aryl iodide **41** and alkyne **1**. Although it has been previously reported that this reaction is unsuccessful^22^ the reaction did furnish acid **42**, albeit in low yield. The hydroxyl linker P2 motif (**43**) required for the ester synthesis was made as described previously.^37^ The linker **43** was then coupled to the indolyl carboxylic acid **42** using either DIC or TBTU/HOAT, with the latter coupling conditions giving a cleaner product (**44**).

The melamine-linker-amine moiety (**45**) required for the synthesis of the corresponding amide analogue (**46**) was made in an analogous fashion to the preparation of **43**.^37^ The amide linkage to the indolyl quinol was attempted using carbodiimide coupling. However, it did not prove possible to separate the amide product (**46**) from a structurally related side product of similar polarity. To avoid the formation of side products, an alternative synthetic strategy was used, where the amide bond was formed to give aryl iodide **47** prior to the introduction of the quinol motif by reaction with **1** (Scheme 4). This same strategy has previously been employed as a means to prepare a small collection of quinol-containing amides.^22^

### 2.4 Biological results

The melamine and benzamidine derived compounds (**35**, **38**, **44**, **46**) were evaluated against *T. brucei* and MRC5 cells (Table 2).

The triazole derivatives that include a targeting motif (**35** and **38**) were found to have increased selectivity for *T. brucei* over mammalian cells, as compared to the potent triazole analogues in the initial screen (**8**–**10**). However, **35** and **38** show a significantly reduced activity compared to the analogues without a targeting group: compounds **8** and **9** have an EC_50_ of 0.13 and 0.16 μM, respectively, compared to **35** and **38** at 11 and 6.2 μM. The reason for this is not known, but may be because of the targeting motif reducing the ability of the quinol moiety to interact with its molecular target(s). Interestingly, **11** and **12** also show the same pattern of improved selectivity and decreased potency. This indicates that it might not be the melamine or benzamidine targeting motif that is conferring selectivity, but some additional factor(s).

The indolyl-sulphonamide derivative **46** also showed improved selectivity compared to analogues without a targeting motif (see compounds **13**–**25**), although its potency is still significantly lower than other indolyl-sulphonamide quinols (see compounds **13**–**25**). However it should be noted that the free acid **42** also shows improved selectivity. This indicates that the melamine moiety may not be responsible for conferring the selectivity observed for **46**. The ester linked indolyl quinol **44** also lacks selectivity. However, it displays better potency than other P2 linked compounds, but is still an order of magnitude less potent than the indolyl quinols with no P2 motif (**13**–**25**).

In order to determine if analogues **35**, **38**, **44** and **46** are substrates for the P2 transporter they were assayed against a line of *T. brucei* lacking the gene which codes for the P2 transporter (Table 2).^33^ If the analogues are transported they would be expected to show greater potency against wild type *T. brucei* than against the P2 knock out line. Compounds **8** and **19**,^25^ which contain the triazole and indolyl quinol pharmacophore respectively, but lack a P2 motif, served as negative controls. Berenil, which is already known to be selectively taken up by the P2 transporter was used as a positive control.^40^ The protocol used to assay the AT1 knock out line is different to the HTS method used to measure the EC_50_ values reported in Table 1, Table 2, therefore the compounds were assayed against wild type *T. brucei* in parallel using the same protocol to provide directly comparable inhibition values.

**Table 1:** Quinol analogues and their inhibitory activities against *T. brucei* and MRC5 cells

**Figure:**
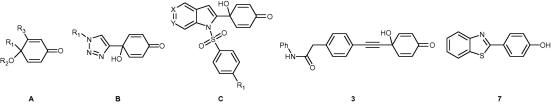

Core	Compd	Structure					EC_50_*T. brucei* (μM)^a^ (SD, *n*)	EC_50_ MRC5 (μM)^a^ (SD, n)	Spec. select^b^	log *P*^c^
		R_1_	R_2_	R_3_	X	Y				
A	**1**	Ethynyl	H	H	n/a	n/a	2.0 (0.80, 4)	2.1 (0.15, 4)	1	0.76
A	**2**	Phenyl	H	H	n/a	n/a	0.75^d^	1.7^d^	2	1.3
n/a	**3**	n/a	n/a	n/a	n/a	n/a	2.2 (0.0017, 2)	0.67 (0.088, 2)	0.3	3.3

	*Benzothiazoles*									
A	**4**^e^	2-Benzo[*d*]thiazole	H	H	n/a	n/a	0.077^d^	0.18^d^	2	1.8
A	**5**	2-Benzo[*d*]thiazole	CH_3_	H	n/a	n/a	0.33^d^	1.1^d^	3	2.7
A	**6**	2-Benzo[*d*]thiazole	H	Cl	n/a	n/a	0.046 (0.0067, 2)	0.055 (0.037, 4)	1	1.9
n/a	**7**	n/a	n/a	n/a	n/a	n/a	>50^d^	>50^d^	n/a	3.5

	*Triazoles*									
B	**8**	4-Methoxyphenyl	n/a	n/a	n/a	n/a	0.13^d^	0.31^d^	2	0.75
B	**9**	4-Methoxybenzyl	n/a	n/a	n/a	n/a	0.16 (0.054, 4)	0.45 (0.12, 4)	3	1.1
B	**10**	6-Benzo[*d*]thiazole	n/a	n/a	n/a	n/a	0.027 (0.0018, 2)	0.069 (0.017, 4)	3	1.5
B	**11**	1-(β-d-Galactopyranosyl, 2,3,4,6-tetraacetate)	n/a	n/a	n/a	n/a	10 (6.2, 4)	>50 (n/a, 3)	>5	−0.30
B	**12**	CH_2_CH_2_CONHPh	n/a	n/a	n/a	n/a	2.7 (0.084, 2)	18 (2.0, 2)	7	0.77

	*Indolyls*									
C	**13**	H	n/a	n/a	CH	CF	0.10^d^	0.17^d^	2	2.3
C	**14**	H	n/a	n/a	CH	CH	0.053^d^	0.11^d^	2	2.3
C	**15**	H	n/a	n/a	N	CH	0.022 (0.0026, 4)	0.041 (0.021, 4)	2	1.4
C	**16**	CH_3_	n/a	n/a	CH	N	0.026 (0.0065, 4)	0.044 (0.028, 4)	2	1.8
C	**17**	NHCO_2_Et	n/a	n/a	CH	CH	0.050 (0.016, 4)	0.086 (0.029, 4)	2	2.3
C	**18**	CH_2_CH_2_NHAc	n/a	n/a	CH	CH	0.012 (0.0019, 2)	0.036 (0.0064, 4)	3	1.9
C	**19**	CH_2_CH_2_CO_2_CH_3_	n/a	n/a	CH	CH	0.16 (0.066, 14)	0.28 (0.14, 13)	2	2.4
C	**20**	CH_2_CH_2_CH_2_OH	n/a	n/a	CH	CH	0.024 (0.0017, 2)	0.051 (0.015, 4)	2	2.1
C	**21**	CH_2_CH_2_CO-*N*-morpholine	n/a	n/a	CH	CH	0.036 (0.0097, 4)	0.067 (0.014, 4)	2	1.5
C	**22**	CH_2_CH_2_CO-4-(1-methylpiperazine)	n/a	n/a	CH	CH	0.018 (0.0032, 6)	0.052 (0.022)	3	1.6
C	**23**	CH_2_CH_2_CH_2_-*N*-morpholine	n/a	n/a	CH	CH	0.026 (0.00042, 2)	0.079 (0.043, 4)	3	2.5
C	**24**	CH_2_CH_2_CH_2_-4-(1-methylpiperazine)	n/a	n/a	CH	CH	0.016 (0.0063, 4)	0.050 (0.023, 4)	3	2.6
C	**25**	SO_2_CH_3_	n/a	n/a	CH	CH	0.024 (0.0017, 4)	0.039 (0.0072, 4)	2	1.5

n/a is not applicable.

a The EC_50_ values are the arithmetic mean of independent determinations with the standard deviations (SD) followed by the number of repeats (*n*) given in parentheses.

b Species selectivity (Spec. select.) is defined as EC_50_ (MRC5)/EC_50_ (*T. brucei*).

c Calculated log *P* values generated using the software package StarDrop by Optibrium.^24^

d Data taken from Konig et al.^21^

e Previously published as PMX 464.

**Table 2:** Activity of targeted compounds against blood stream form *T. b. brucei*, MRC5 cells and a P2 double knock out *T. brucei* cell line (*T. brucei* AT1 KO)

Compd	*T. brucei* EC_50_ (μM)^a^^,^^b^ (SD, *n*)	MRC5 EC_50_ (μM) (SD, *n*)	Spec. select.^c^	*T. brucei* EC_50_ (μM)^d^^,^^e^ (SD)	*T. brucei* AT1 KO EC_50_ (μM)^d^^,^^e^ (SD)	Cell line selectivity^f^	log *D*^h^	log *P*^h^
**8**	0.13^g^	0.31^g^	2.4	0.095 (0.01)	0.074 (0.08)	0.78	0.76	0.76
**35**	11 (0.39, 4)	>50 (n/a, 2)	>5	5.0 (0.61)	6.0 (0.61)	1.2	−1.2	0.04
**38**	6.2 (0.50, 4)	>50 (n/a, 2)	>8	3.8 (0.4)	3.8 (0.4)	1.0	−0.66	−0.47
**19**	0.16 (0.066, 14)	0.28 (0.14, 13)	1.8	0.060 (0.002)	0.080 (0.002)	1.3	2.4	2.4
**42**	0.74 (0.027, 4)	3.3 (0.39, 4)	4.5	ND	ND	—	−0.30	2.1
**44**	0.35 (0.12, 7)	0.95 (0.24, 6)	2.7	0.25 (0.026)	0.26 (0.026)	1.0	1.5	1.8
**46**	3.2 (0.36, 7)	18 (1.2, 6)	5.6	1.7 (0.17)	1.8 (0.17)	1.1	1.4	1.3
Berenil	ND	ND	—	0.031 (0.0028)	0.33 (0.0090)	11	1.8	0.00

ND is not determined.

a Determined using a 96-well HTS assay as previously described.^38^

b The EC_50_ values are the arithmetic mean of independent determinations, with the standard deviations (SD) followed by the number of repeats (*n*) given in parentheses.

c Species selectivity (Spec. select.) is defined as EC_50_ (MRC5)/EC_50_ (*T. brucei*).

d Determined using a 96-well manual assay as previously described.^39^

e The EC_50_ values are the weighted mean of three independent experiments, the standard deviations (SD) are given in parentheses.

f Cell line selectivity is defined as EC_50_ (AT1 KO)/EC_50_ (WT).

g Data taken from König et al.^21^

h Calculated using StarDrop.^24^

The results in Table 2 show that there is no difference in the EC_50_s for analogues **35**, **38**, **44** and **46** in wild type *T. brucei* compared with those for the P2 knock out strain. These analogues show identical selectivity (∼1) to the negative controls (**8** and **19**) and are therefore not substrates for the P2 transporter protein. This indicates the compounds are unlikely to be actively transported by the parasite. Berenil shows a 10-fold selectivity for the wild type strain, consistent with values measured in the literature for this cell line, confirming the validity of this assay data.

Because the transporter is not fully characterised, it is open to speculation why these motifs do not allow the active transport of these compounds. It is possible that the indolyl quinols (**44** and **46**) are too large (MW >600) to be channelled through transporters whose physiological substrates are much smaller (e.g. adenine, MW = 135). It is known that a benzamidine moiety needs to have an oxygen or nitrogen atom in the *para*-position in order to be a substrate for the P2 transporter. However, it is not known if a *para*-triazole nitrogen (as is the case in analogue **35**) provides a suitable atom to complete the recognition motif; this may explain why **35** is not actively transported. Similarly, it has not been determined if a triazole-melamine bi-aryl system (as in **38**) can act as a substrate for the P2 transporter in an analogous fashion to a phenyl-melamine system.

Assuming the compounds are predominantly taken up by passive diffusion, there could be a correlation between the log *P* value and the activity against *T. brucei*. Thus compounds **35** and **38** have low log *P* values, which may correlate to poor passive diffusion across the cell membrane. The other compounds have larger log *P* values, implying they are more lipophilic and hence have greater cellular permeability. The only exception to this is berenil, which has a low log *P* value, yet is very active against *T. brucei*; berenil is known to be taken up rapidly by the P2 transporter. However this permeability hypothesis cannot explain the lack of activity of compounds **35** and **38** against MRC5 cells. It is possible that another transporter, such as HAPT1 or LAPT1^33^ may be involved in weak uptake of these compounds in *T. brucei*.

### 2.5 Summary

Screening a collection of quinols established that this inhibitor class is very potent against *T. brucei*, although the counter-screen against MRC5 cells showed they lack sufficient selectivity to be of therapeutic value against these parasites. The strategy of synthesising compounds incorporating a P2 targeting motif demonstrated it was possible to improve their selectivity, but this proved to be at the expense of potency. Further experiments using P2 knock out parasites confirmed that the P2 motif-containing compounds are not substrates for the P2 transporter; therefore they derive their selectivity through another mechanism.

## 3 Experimental section

### 3.1 Biology

#### 3.1.1 Cell-based assays

The 96-well HTS assays against blood stream form *T. brucei*^38^ and MRC5 cells were carried out as reported previously.^39^ The manual assays performed on the AT1 double knock out *T. brucei* and wild type *T. brucei* were conducted as described by Jones et al.^39^

### 3.2 Chemistry

General: Chemicals and solvents were purchased from the Aldrich Chemical Company, Fluka, ABCR, VWR, Acros, Fisher Chemicals and Alfa Aesar and were used as received unless otherwise stated. Air and moisture sensitive reactions were carried out under an inert atmosphere of argon in oven-dried glassware. Petrol refers to the 40–60 °C boiling fraction. Analytical thin-layer chromatography (TLC) was performed on pre-coated TLC plates (layer 0.20 mm Silica Gel 60 with fluorescent indicator UV254) (Merck). Developed plates were air-dried and analysed under a UV lamp (UV254/365 nm), and where necessary, stained with a solution of ninhydrin or iodine on silica to aid identification. ^1^H, ^13^C NMR and 2D-NMR spectra were recorded on a Bruker Avance DPX 500 spectrometer (^1^H at 500.1 MHz, ^13^C at 125.8 MHz). Chemical shifts (*δ*) are expressed in ppm recorded using the residual solvent as the internal reference in all cases. Signal splitting patterns are described as singlet (s), doublet (d), triplet (t), quartet (q), multiplet (m), broad (br), or a combination thereof. Coupling constants (*J*) are quoted to the nearest 0.5 Hz. LC–MS analyses were performed with either an Agilent HPLC 1100 series connected to a Bruker Daltonics MicrOTOF, or an Agilent Technologies 1200 series HPLC connected to an Agilent Technologies 6130 quadrupole LC/MS, both instruments were connected to an Agilent diode array detector. High resolution electrospray measurements were performed on a Bruker Daltonics MicrOTOF mass spectrometer [*Caution: take appropriate care when preparing and handling azides.*].

#### 3.2.1 Screening compounds

Compounds **2**, **4**–**11**, **13**–**18** and **20**–**25** were provided by Dr Andrew McCarroll (Pharminox Ltd, Nottingham, UK) and were prepared as previously described.^22^, ^23^

#### 3.2.2 Synthesis

##### 3.2.2.1 4,4-Dimethoxycyclohexa-2,5-dienone (**27**)

Compound previously reported by Pelter and Elgendy^31^: 4-methoxy phenol (**26**) (2.48 g, 20 mmol) was dissolved in methanol (40 mL) and a solution of PIDA (6.44 g, 20 mmol) in methanol (100 mL) was transferred by cannula over 1.66 h. The reaction mixture was concentrated in vacuo then the crude purified by column chromatography (DCM/petrol 0:100–100:0) to afford the product as a yellow oil (2.35 g, 76%). ^1^H NMR (500 MHz, CDCl_3_): *δ* = 3.23 (s, 6H, 2 × CH_3_), 5.94 (d, 2H, *J* = 10.5 Hz, d, 2 × CH), 6.52 ppm (d, 2H, *J* = 10.5 Hz, 2 × CH); ^13^C NMR (125 MHz, CDCl_3_) *δ* = 50.2 (CH_3_), 129.7 (CH), 92.3 (C), 143.3 (CH), 184.9 ppm (C).

##### 3.2.2.2 4-Ethynyl-4-hydroxycyclohexa-2,5-dienone (**1**)

Compound **27** (2.35 g, 15.2 mmol) was dissolved in THF (100 mL) and cooled to −78 °C. Ethynylmagnesium bromide (60.8 mL, 0.5 M solution in THF, 30.4 mmol), was added slowly by syringe. The mixture was allowed to warm to room temperature, and was stirred for 2 h. The reaction mixture was diluted with ether, quenched with saturated aqueous NH_4_Cl solution, and then extracted with Et_2_O (3 × 150 mL). The combined organic extracts were then washed with water, brine, dried over MgSO_4_, and filtered. They were then concentrated in vacuo, and the crude was redissolved in chloroform and stirred with silica gel overnight to afford complete deprotection of the ketone. The solution was again filtered and concentrated then purified by column chromatography (EtOAc/hexane 25:75–60:40) to afford the product as sticky yellow oil (1.86 g, 90%). ^1^H NMR (500 MHz, CDCl_3_) *δ* = 2.64 (s, 1H, CH), 6.22 (d, 2H, *J* = 10.1 Hz, 2 × CH), 6.94 ppm (d, 2H, *J* = 10.1 Hz, 2 × CH); ^13^C NMR (125 MHz, CDCl_3_) *δ* = 62.0 (CH), 74.5 (C), 80.3 (C), 127.3 (CH), 147.1 (CH), 185.2 ppm (C); LRMS (LC–MS ES^+^): *m*/*z* 135 (M+H^+^, 100); HRMS: calcd mass for C_8_H_7_O_2_ [M+H^+^]: 135.0441, found 135.0443 (−2.05 ppm).

##### 3.2.2.3 Synthesis of 3-bromo-*N*-phenylpropanamide (**29**)^41^

3-Bromopropionyl chloride (2.2 mmol, 377 mg) was slowly added to a solution of aniline (2 mmol, 186 mg) in anhydrous CH_2_Cl_2_ (60 mL) at 0 °C. Pyridine (2.2 mmol, 174 mg) was added to the resultant mixture and the reaction was warmed to 25 °C with stirring for 1 h. Workup was initiated by the addition of a NaHCO_3_ solution (satd aq 40 mL), the layers separated and the CH_2_Cl_2_ layer further extracted with NaHCO_3_ (satd aq 40 mL), water (2 × 40 mL) and brine (40 mL). The CH_2_Cl_2_ layer was dried over MgSO_4_, filtered and the solvent removed under reduced pressure. The crude product was purified by flash column chromatography (EtOAc/Hexane 0:100–50:50) to give the product amide as a white solid (158 mg, 69%) contaminated with ∼15% of the olefin from the elimination of HBr. ^1^H NMR (500 MHz, CDCl_3_) *δ* = 2.95 (t, 2H, *J* = 6.5 Hz, CH_2_), 3.72 (t, 2H, *J* = 6.5 Hz, CH_2_), 7.13 (t, 1H, *J* = 7.5 Hz, ArH), 7.28 (br s, 1H, NH), 7.34 (t, 2H, *J* = 7.5 Hz 2 × ArH), 7.52 ppm (d, 2H, *J* = 7.5 Hz, 2 × ArH); ^13^C NMR (125 MHz, CDCl_3_) *δ* = 27.2 (CH_2_), 40.7 (CH_2_), 120.2 (CH), 124.8 (CH), 129.1 (CH), 137.4 (C), 168.1 ppm (C); LRMS (LC–MS ES^+^): *m*/*z* 228 (^79^Br M+H^+^, 100%), 230 (^81^Br M+H^+^, 100%).

##### 3.2.2.4 Synthesis of 3-azido-*N*-phenylpropanamide (**30**)^41^

Sodium azide (0.76 mmol, 50 mg) was added to a solution of alkyl bromide **28** (0.69 mmol, 158 mg) in anhydrous DMF (2 mL) and the reaction heated at 60 °C with stirring for 16 h. Subsequently the reaction was diluted with EtOAc (10 mL), successively washed with NaHCO_3_ (satd aq 1 × 10 mL), brine (1 × 10 mL), dried over MgSO_4_, filtered and the EtOAc removed under reduced pressure. The crude product was purified by silica flash column chromatography (EtOAc/Hexane 0:100–50:50) to give the product azide as a white waxy solid (87 mg, 69%) contaminated with olefin carried through from the previous reaction. ^1^H NMR (500 MHz, CDCl_3_) *δ* = 2.60 (t, 2H, *J* = 6.5 Hz, CH_2_), 3.72 (t, 2H, *J* = 6.5 Hz, CH_2_), 7.13 (t, 1H, *J* = 7.5 Hz, ArH), 7.33 (t, 2H, *J* = 7.5 Hz 2 × ArH), 7.45 (br s, 1H, NH), 7.51 ppm (d, 2H, *J* = 7.5 Hz, 2 × ArH); ^13^C NMR (125 MHz, CDCl_3_ *δ* = 36.8 (CH_2_), 47.3 (CH_2_), 120.2 (CH), 124.7 (CH), 129.1 (CH), 137.5 (C), 168.4 ppm (C); LRMS (LC–MS ES^+^): *m*/*z* 120 (M−CH_2_CH_2_N_3_^+^, 30%), 163 (M+H−N_2_^+^, 53%), 191 (M+H^+^, 100%), 213 (M+Na^+^, 14%).

##### 3.2.2.5 3-(4-(1-Hydroxy-4-oxocyclohexa-2,5-dien-1-yl)-1*H*-1,2,3-triazol-1-yl)-*N*-phenylpropanamide (**12**)^36^

Copper (I) iodide (0.025 mmol, 5 mg) was added to a solution of azide **29** (0.25 mmol, 48 mg) and alkyne **1** (0.3 mmol, 34 mg) in degassed CHCl_3_ (2 mL) at 0 °C, followed by the slow addition of 2,6-lutidine (0.3 mmol, 32 mg). The resultant reaction mixture was allowed to warm to 25 °C and stirred for 16 h, before being diluted with CH_2_Cl_2_ (2 mL) and washed with NH_4_Cl solution (satd aq 4 mL). The aqueous was then back-extracted with CH_2_Cl_2_ (3 × 10 mL), the organic layers combined, dried over MgSO_4_, filtered and the solvent removed under reduced pressure. The crude product was purified by silica flash column chromatography (MeOH/EtOAc/Hexane 0:0:100–10:90:0) to give the product 1,2,3-triazole as an off-white foam (44 mg, 54%). ^1^H NMR (500 MHz, DMSO-*d*_6_) *δ* = 2.99 (t, 2H, *J* = 7.0 Hz, CH_2_), 4.66 (t, 2H, *J* = 7.0 Hz, CH_2_), 6.13–6.16 (m, 2H, AA′BB′, 2 × CH), 6.47 (s, 1H, OH), 7.05 (t, 1H, *J* = 7.5 Hz, ArH), 7.12–7.15 (m, 2H, AA′BB′, 2 × CH), 7.28–7.32 (m, 2H, 2 × ArH), 7.55 (d, 2H, *J* = 8.0 Hz, ArH), 8.17 (s, 1H, ArH), 10.03 ppm (s, 1H, NH); ^13^C NMR (125 MHz, DMSO-*d*_6_) *δ* = 36.2 (CH_2_) 45.7 (CH_2_), 65.2 (C), 119.1 (CH), 123.1 (CH), 123.3 (CH), 126.0 (CH), 128.7 (CH), 138.9 (C), 147.8 (C), 150.7 (CH), 168.0 (C), 185.2 ppm (C); LRMS (LC–MS ES+): *m*/*z* 325 (M+H^+^, 100%), 649 (2M+H^+^, 36%); HRMS (ES^+^): calcd for C_17_H_16_N_4_Na_1_O_3_ [M+Na]^+^ 347.1115, found 347.1127 (−3.63 ppm).

##### 3.2.2.6 2-(4-((1-Hydroxy-4-oxocyclohexa-2,5-dien-1-yl)ethynyl)phenyl)acetic acid (**32**)^25^

Copper (I) iodide (0.1 mmol, 19 mg) and tetrakis(triphenylphosphine)palladium(0) (0.06 mmol, 69 mg) were added to a solution of 4-iodophenylacetic acid (**31**) (1.16 mmol, 304 mg) and alkyne **1** (1 mmol, 134 mg) in degassed dimethylacetamide (DMAc) (1 mL), *^i^*Pr_2_NH (200 μL) and water (40 μL) and the resultant mixture heated under microwave irradiation at 100 °C for 10 min. After cooling the reaction mixture was added to CH_2_Cl_2_/water (1:1, 200 mL), the layers separated and the aqueous layer extracted with CH_2_Cl_2_ (3 × 50 mL). The combined CH_2_Cl_2_ layers were dried over MgSO_4_, filtered and the solvent removed under reduced pressure to give a brown tar, which was purified by silica flash column chromatography (MeOH/CH_2_Cl_2_ 0:100–20:80) to give the product as its *^i^*Pr_2_NH salt. The salt was partitioned between 1 N aqueous HCl/EtOAc (1:1, 10 mL), the layers separated, the EtOAc layer dried over MgSO_4_, filtered and evaporated under reduced pressure to give the free acid as a brown solid (29 mg, 11%). ^1^H NMR (500 MHz, CD_3_OD) *δ* = 3.52 (s, 2H, CH_2_), 6.06–6.10 (m, 2H, AA′BB′, 2 × CH), 6.93–6.96 (m, 2H, AA′BB′, 2 × CH), 7.17–7.19 (m, 2H, AA′BB′, 2 × ArH), 7.28–7.31 ppm (m, 2H, AA′BB′, 2 × ArH); LRMS (LC–MS ES^+^): *m*/*z* 251 (M−OH^+^, 100%), 269 (M+H^+^, 89%).

##### 3.2.2.7 2-(4-((1-Hydroxy-4-oxocyclohexa-2,5-dien-1-yl)ethynyl)phenyl)-*N*-phenylacetamide (**3**)

DIPEA (0.4 mmol, 52 mg) was added to a solution of acid **32** (0.11 mmol, 29 mg), aniline (0.1 mmol, 9 mg) and PyBrop (0.1 mmol, 47 mg) in anhydrous DMF (500 μL) at 0 °C. The reaction was allowed to warm to 25 °C and stirred for 16 h before being diluted with EtOAc (10 mL) and washed with NaHCO_3_ solution (satd aq 50 mL). The aqueous layer was back-extracted with EtOAc (3 × 50 mL), the combined EtOAc layers were dried over MgSO_4_, filtered and the solvent removed under reduced pressure. The crude product was purified by silica flash column chromatography (MeOH/CH_2_Cl_2_ 0:100–20:80) to give the product as a brown solid (7 mg, 20%). ^1^H NMR (500 MHz, DMSO-*d*_6_) *δ* = 3.36 (s, 1H, OH), 3.73 (s, 2H, CH_2_), 6.20–6.23 (m, 2H, AA′BB′, 2 × CH), 6.85 (s, 1H, ArH), 7.08–7.13 (m, 3H, 2 × CH & ArH), 7.35 (dd, 2H, *J* = 7.5, 7.5 Hz, 2 × ArH), 7.39–7.42 (m, 2H, AA′BB′, 2 × ArH), 7.46–7.48 (m, 2H, AA′BB′, 2 × ArH), 7.64 (d, 2H, *J* = 7.5 Hz, 2 × ArH), 10.22 ppm (br s, 1H, NH); ^13^C NMR (125 MHz, DMSO-*d*_6_) *δ* = 43.0 (CH_2_), 61.6 (C), 83.9 (C), 86.9 (C), 119.1 (CH), 119.3 (C), 123.3 (CH), 125.7 (CH), 128.7 (CH), 129.6 (CH), 131.5 (CH), 137.4 (C), 139.1 (C), 148.5 (CH), 168.5 (C), 184.5 (C) ppm. MS (LC–MS ES+): *m*/*z* 326 (M−OH^+^, 16%), 344 (M+H^+^, 100%), 366 (M+Na^+^, 46%); HRMS (ES^+^): calcd for C_22_H_18_N_1_O_3_ [M+H]^+^ 344.1281, found 344.1281 (0.15 ppm).

##### 3.2.2.8 Methyl 3-(4-((2-(1-hydroxy-4-oxocyclohexa-2,5-dien-1-yl)-1*H*-indol-1-yl)sulfonyl)phenyl) propanoate (**19**)^25^

Copper (I) iodide (0.1 mmol, 19 mg) and tetrakis(triphenylphosphine) palladium(0) (0.06 mmol, 69 mg) were added to a solution of aryl iodide **40** (1.16 mmol, 517 mg) and alkyne **1** (1.0 mmol, 134 mg) in degassed DMAc (1.0 mL), *^i^*Pr_2_NH (200 μL) and water (40 μL) and the resultant mixture heated under microwave irradiation at 100 °C for 10 min. After cooling the reaction mixture was added to CH_2_Cl_2_/H_2_O (1:1, 200 mL), the layers separated and the aqueous extracted with CH_2_Cl_2_ (3 × 50 mL). The combined CH_2_Cl_2_ layers were dried over MgSO_4_, filtered and the solvent removed under reduced pressure. The product was purified by silica flash column chromatography (EtOAc/hexane 0:100–50:50) to give the product as a brown solid (314 mg, 70%). ^1^H NMR (500 MHz, CDCl_3_): *δ* 2.58 (t, 2H, *J* = 7.5 Hz, CH_2_), 2.93 (t, 2H, *J* = 7.5 Hz, CH_2_), 3.61 (s, 3H, CH_3_), 5.48 (s, 1H, OH), 6.32–6.29 (m, 2H, AA′BB′ 2 × CH), 6.80 (s, 1H, ArH), 7.32–7.20 (m, 4H, 4 × ArH), 7.43 (d, 1H, *J* = 7.5 Hz, ArH), 7.58–7.55 (m, 2H, AA′BB′ 2 × CH), 7.80–7.78 (m, 2H, AA′BB′ 2 × ArH), 8.00 ppm (d, 1H, *J* = 8.5 Hz, ArH); ^13^C NMR (125 MHz, CDCl_3_): *δ* 30.6 (CH_2_), 34.6 (CH_2_), 51.8 (CH_3_), 67.6 (C), 113.6 (CH), 115.2 (CH), 121.7 (CH), 124.6 (CH), 126.2 (CH), 126.9 (CH), 127.6 (CH), 128.3 (C), 129.3 (CH), 135.4 (C), 138.2 (C), 140.7 (C), 147.6 (CH), 147.8 (C), 172.5 (C), 185.0 ppm (C); MS (LC–MS ES^+^): *m*/*z* 434 (M−OH^+^, 85%), 452 (M+H^+^, 100%), 474 (M+Na^+^, 33%). HRMS (ES+): calcd for C_24_H_21_N_1_Na_1_O_6_S_1_ [M+Na]^+^ 474.0982, found 474.0977 (1.01 ppm).

##### 3.2.2.9 4-Azidobenzimidamide sulfate (**34**)

Compound **33** (500 mg, 3.7 mmol) was dissolved in H_2_SO_4_, and the viscous yellow-green solution was cooled to 0 °C. NaNO_2_ (200 mg, 2.9 mmol) was added in two portions and stirred at 0 °C for 30 min. NaN_3_ (200 mg, 3.08 mmol) was added along with one drop of water. The reaction mixture was stirred overnight, and turned from a yellow solution to a thick orange suspension. The reaction mixture was filtered and the pale orange crystals washed with H_2_O and MeOH. The orange crystals were then dried (332 mg, 56%). ^1^H NMR (500 MHz, DMSO-*d*_6_) *δ* = 7.24 (d, 2H, *J* = 9.0 Hz, 2 × ArH), 7.73 (d, 2H, *J* = 9.0 Hz, 2 × ArH), 8.80 (s, 2H, NH_2_), 9.17 ppm (s, 2H, NH_2_). ^13^C NMR (125 MHz, DMSO-*d*_6_) *δ* = 119.6 (CH), 124.1 (C), 130.1 (CH), 145.2 (C), 164.4 ppm (C); LRMS (LC–MS ES^+^): *m*/*z* 162 (M+H^+^, 100%); HRMS: calcd for C_7_H_7_N_5_ [M+H]^+^ 162.0774, found 162.0768 (4.01 ppm).

##### 3.2.2.10 4-(4-(1-Hydroxy-4-oxocyclohexa-2,5-dienyl)-1*H*-1,2,3-triazol-1-yl)benzimidamide (**35**)

Compound **1** (60 mg, 0.447 mmol), compound **34** (60 mg, 0.373 mmol) and CuI (7 mg, 0.037 mmol) were dissolved in dry, degassed MeCN (1 mL). 2,6-Lutedine (52 μL, 0.447 mmol) was added at 0 °C and the reaction mixture was stirred for 18 h under argon. The crude was concentrated then purified twice by semi-prep HPLC under basic conditions, to afford the product as a brown powder (5 mg, 4%). ^1^H NMR (500 MHz, CD_3_OD) *δ* = 6.30 (d, 2H, *J* = 10.0 Hz, 2 × CH), 7.23 (d, 2H, *J* = 10.0 Hz, 2 × CH), 8.2 (d, 2H, *J* = 8.0 Hz, 2 × ArH), 8.20 (d, 2H, *J* = 8.4 Hz, 2 × ArH), 8.84 ppm (s, 1H, ArH); LRMS (LC–MS ES^+^): *m*/*z* 296 (M+H^+^, 100%); HRMS: calcd for C_15_H_14_N_5_O_2_ [M+H]^+^: 296.1142, found 296.1130 (4.18 ppm).

##### 3.2.2.11 6-Azido-1,3,5-triazine-2,4-diamine (**37**)

Compound **36** (500 mg, 3.44 mmol) was suspended in DMF (3 mL), and TEA (275 μL, 2.06 mmol) was added. NaN_3_ (270 mg, 4.15 mmol) was added in H_2_O (2 mL) and the reaction was heated to 115 °C for 18 h. During heating a precipitate formed which was filtered off and washed with water, methanol and DCM to afford the product as a beige powder (160 mg, 31%). ^1^H NMR (500 MHz, DMSO-*d*_6_) *δ* = 6.91 (br s, 2H, NH_2_), 6.97 ppm (br s, 2H, NH_2_); ^13^C NMR (125 MHz, DMSO-*d*_6_) *δ* = 167.6 (C), 168.4 ppm (C); LRMS (LC–MS ES^+^): *m*/*z* 153 (M+H^+^, 100%); HRMS: calcd for C_3_H_4_N_8_ [M+H]^+^: 153.0632, found 153.0645 (−8.45 ppm).

##### 3.2.2.12 4-(1-(4,6-Diamino-1,3,5-triazin-2-yl)-1*H*-1,2,3-triazol-4-yl)-4-hydroxycyclohexa-2,5-dienone (**38**)

Compound **1** (72 mg, 0.474 mmol), compound **26** (60 mg, 0.395 mmol) and CuI (8 mg, 0.040 mmol) were dissolved in dry, degassed MeCN (1 mL). 2,6-Lutedine (55 μL, 0.474 mmol) was added at 0 °C, and the reaction mixture was stirred for 18 h under argon. The crude was concentrated then purified twice by semi-prep HPLC under basic conditions, to afford the product as an off-white powder (4 mg, 3%). ^1^H NMR (500 MHz, MeOD) *δ* = 6.29 (d, 2H, *J* = 10.0 Hz, 2H, 2 × CH), 7.19 (d, 2H, *J* = 10.0 Hz, 2 × CH), 8.75 ppm (s, 1H, CH); ^13^C NMR (125 MHz, CDCl_3_) *δ* = 67.0 (C), 122.3 (CH), 128.3 (CH), 150.1 (C), 151.1 (CH), 162.3 (C), 169.5 (C), 187.2 ppm (C); LRMS (LC–MS ES^+^): *m*/*z* 287 (M+H^+^, 100%); HRMS: calcd for C_11_H_11_N_8_O_2_ [M+H]^+^: 287.0999, found 287.0990 (3.31 ppm).

##### 3.2.2.13 3-(4-(*N*-(2-Iodophenyl)sulfamoyl)phenyl)propanoate (**40**)^22^

2-Iodoaniline (20 mmol, 4.38 g) was added to a solution of methyl 3-(4-(chlorosulfonyl)phenyl)propanoate (**39**) (20 mmol, 5.25 g) in anhydrous THF/pyridine (1:1, 16 mL) and the reaction mixture stirred at 25 °C for16 h before removal of the solvent under reduced pressure. The resultant crude product was redissolved in CHCl_3_ (50 mL), washed with citrate solution (10% w/v aq 3 × 50 mL), dried over MgSO_4_, filtered and the CHCl_3_ removed under reduced pressure to give an orange semi-solid which was further purified by recrystallisation from hot EtOAc/petrol to give a white crystalline solid (5.54 g, 62%). ^1^H NMR (500 MHz, CDCl_3_) *δ* = 2.64 (t, 2H, *J* = 7.5 Hz, CH_2_), 3.00 (t, 2H, *J* = 7.5 Hz, CH_2_), 6.78 (br s, 1H, NH), 6.86 (ddd, 1H, *J* = 8.0, 7.5, 1.5 Hz, ArH), 7.26–7.29 (m, 2H, AA′BB′, 2 × ArH), 7.32–7.35 (m, 1H, ArH), 7.66–7.69 ppm (m, 4H, 4 × ArH); ^13^C NMR (125 MHz, DMSO-*d*_6_) *δ* = 30.0 (CH_2_), 34.3 (CH_2_), 51.3 (CH_3_), 98.6 (C), 126.9 (CH), 127.1 (CH), 128.4 (CH), 128.9 (CH), 129.0 (CH), 138.2 (C), 138.5 (C), 139.6 (CH), 145.9 (C), 172.4 ppm (C); LRMS (LC–MS ES^+^): *m*/*z* 446 (M+H^+^, 94%), 463 (M+H_2_O^+^, 100%).

##### 3.2.2.14 Synthesis of 3-(4-(*N*-(2-iodophenyl)sulfamoyl)phenyl)propanoic acid (**41**)^22^

Ester **40** (1.12 mmol, 500 mg) was dissolved in KOH solution (10% w/v, aq 10 mL) and heated at 100 °C for 30 min. The reaction was then cooled to 0 °C before adjusting the mixture to pH 2 by addition of HCl (1 N, aq). After standing at 0 °C for 1 h the resulting precipitate was collected by filtration, washed with water (2 × 10 mL) and dried under vacuum to give the product acid as a white solid (459 mg, 95%). ^1^H NMR (500 MHz, DMSO-*d*_6_) *δ* = 2.58 (t, 2H, *J* = 7.5 Hz, CH_2_), 2.90 (t, 2H, *J* = 7.5 Hz, CH_2_), 6.96–6.99 (m, 2H, 2 × ArH), 7.28–7.32 (m, 1H, ArH), 7.42–7.44 (m, 2H, AA′BB′, 2 × ArH), 7.61–7.63 (m, 2H, AA′BB′, 2 × ArH), 7.84 (dd, 1H, *J* = 8..5, 1.5 Hz, ArH), 9.72 (br s, 1H, NH), 12.22 ppm (br s, 1H, COOH); ^13^C NMR (125 MHz, DMSO-*d*_6_) *δ* = 30.1 (CH_2_), 34.6 (CH_2_), 98.7 (CI), 126.8 (CH), 127.1 (CH), 128.4 (CH), 128.9 (CH), 129.0 (CH), 138.2 (C), 138.4 (C), 139.6 (CH), 146.3 (C), 173.5 ppm (C); LRMS (LC–MS ES^+^): *m*/*z* 432 (M+H^+^, 52%), 449 (M+H_2_O^+^, 100%), 880 (2M+H_2_O^+^, 48%).

##### 3.2.2.15 3-(4-((2-(1-Hydroxy-4-oxocyclohexa-2,5-dien-1-yl)-1*H*-indol-1-yl)sulfonyl)phenyl)propanoic acid (**42**)^25^

Copper (I) iodide (0.25 mmol, 48 mg) and tetrakis(triphenylphosphine) palladium(0) (0.15 mmol, 173 mg) were added to a solution of aryl iodide **41** (2.9 mmol, 1.25 g) and alkyne **1** (2.5 mmol, 335 mg) in degassed DMAc (2.5 mL), *^i^*Pr_2_NH (500 μL) and water (100 μL) and the resultant mixture heated under microwave irradiation at 100 °C for 10 min. After cooling the reaction mixture was added to CH_2_Cl_2_/citrate solution (10% w/v aq) (1:1, 200 mL), the layers separated and the aqueous layer extracted with CH_2_Cl_2_ (3 × 50 mL). The combined CH_2_Cl_2_ layers were dried over MgSO_4_, filtered and the solvent removed under reduced pressure to give a brown oil, which was purified by silica flash column chromatography (MeOH/CH_2_Cl_2_ 0:100 to 20:80) to give the product as a pale brown solid (151 mg, 14%). ^1^H NMR (500 MHz, CDCl_3_) *δ* = 2.61 (t, 2H, *J* = 7.5 Hz, CH_2_), 2.94 (t, 2H, *J* = 7.5 Hz, CH_2_), 5.55 (br s, 1H, OH), 6.32–6.35 (m, 2H, AA′BB′, 2 × CH), 6.80 (d, 1H, *J* = 1.0 Hz, ArH), 7.21–7.24 (m, 1H, ArH), 7.28–7.33 (m, 3H, 2 × CH & ArH), 7.56–7.60 (m, 2H, AA′BB′, 2 × ArH), 7.78–7.81 (m, 2H, AA′BB′, 2 × ArH), 8.00 ppm (dd, 1H, *J* = 8.5, 1.0 Hz, ArH); ^13^C NMR (125 MHz, DMSO-*d*_6_) *δ* = 30.0 (CH_2_), 34.1 (CH_2_), 67.1 (C), 112.3 (CH), 115.1 (CH), 121.6 (CH), 123.9 (CH), 125.5 (CH), 126.5 (CH), 126.9 (CH), 128.0 (C), 129.1 (CH), 135.9 (C), 137.8 (C), 141.1 (C), 148.0 (C), 149.8 (CH), 173.4 (C), 185.0 ppm (C); LRMS (LC–MS ES+): *m*/*z* 420 (M−OH^+^, 77%), 438 (M+H^+^, 100%), 897 (2M+Na^+^, 13%); HRMS (ES^+^): calcd for C_23_H_19_N_1_Na_1_O_6_S_1_ [M+Na]^+^ 460.0825, found 460.0826 (−0.06 ppm).

##### 3.2.2.16 3-((4,6-Diamino-1,3,5-triazin-2-yl)amino)propyl 3-(4-((2-(1-hydroxy-4-oxocyclohexa-2,5-dien-1-yl)-1*H*-indol-1-yl)sulfonyl)phenyl)propanoate (**44**)

Compound **42**^37^ (28 mg, 0.154 mmol), compound **43** (45 mg, 0.103 mmol) and DIPEA (20 μL, 0.113 mmol) were dissolved in DMF (0.5 mL). The reaction mixture was cooled to 0 °C and DIC (24 μL, 0.154 mmol) was added. The reaction mixture was stirred under argon for 18 h, and then purified directly by semi-prep HPLC to afford the product as a beige powder (42 mg, 67%). ^1^H NMR (500 MHz, MeOD) *δ* = 1.78 (dd, 2H, *J* = 6.5 Hz, CH_2_), 2.64 (t, 2H, *J* = 7.5 Hz, CH_2_), 2.94 (t, 2H, *J* = 7.5 Hz, CH_2_), 3.27 (t, 2H, *J* = 7.0 Hz, CH_2_), 4.07 (t, 2H, *J* = 6.0 Hz, CH_2_), 6.27 (d, 2H, *J* = 10.0 Hz, 2 × CH), 6.94 (s, 1H, CH), 7.24 (t, 1H, *J* = 7.5 Hz, ArH), 7.34 (d, 2H, *J* = 8.5 Hz, 2 × ArH), 7.51 (d, 1H, *J* = 8.0 Hz, ArH), 7.58 (d, 2H, *J* = 10.0, 2 × CH), 7.84 (d, 2H, *J* = 8.5 Hz, 2 × ArH), 8.20 ppm (d, 1H, *J* = 8.5 Hz, ArH); ^13^C NMR (125 MHz, CDCl_3_) *δ* = 29.8 ppm (CH_2_), 31.6 (CH_2_), 38.3 (CH_2_), 35.7 (CH_2_), 63.6 (CH_2_), 68.7 (C), 114.2 (CH), 116.6 (CH), 122.2 (C), 125.2 (CH), 126.7 (CH), 127.9 (CH), 128.4 (CH), 130.0 (CH), 130.1 (CH), 138.0 (C), 140.2 (C), 142.2 (C), 149.1 (CH), 151.2 (C), 167.5 (C), 168.2 (C), 174.1 (CNH_2_), 187.4 ppm (C); LRMS (LC–MS ES^+^): *m*/*z* 153 (M+H^+^, 100%); HRMS: calcd for C_29_H_29_N_7_O_6_S [M+H]^+^: 604.1973, found 604.1982 (−1.55 ppm).

##### 3.2.2.17 *N*-(3-((4,6-Diamino-1,3,5-triazin-2-yl)amino)propyl)-3-(4-(*N*-(2-iodophenyl)sulfamoyl)phenyl) propanamide (**47**)

Acid **41** (539 mg, 1.25 mmol), amine **45**^37^ (344 mg, 1.875 mmol), TBTU (602 mg, 1.875 mmol) and HOAt (255 mg, 1.875 mmol) were dissolved in anhydrous DMF (20 mL) and cooled to 0 °C, before the addition of DIPEA (485 mg, 3.75 mmol). Subsequently, the reaction mixture was stirred at 25 °C for 18 h followed by purification of half of the material by reverse phase HPLC to afford the product as a white solid (42 mg, 11%). ^1^H NMR (500 MHz, CD_3_OD) *δ* = 1.67 (tt, 2H, *J* = 7.0, 7.0 Hz, CH_2_), 2.52 (t, 2H, *J* = 7.5 Hz, CH_2_), 3.00 (t, 2H, *J* = 7.5 Hz, CH_2_), 3.19 (t, 2H, *J* = 7.0 Hz, CH_2_), 3.28 (t, 2H, *J* = 7.0 Hz, CH_2_), 6.92–6.95 (m, 1H, ArH), 7.31–7.37 (m, 3H, 3 × ArH), 7.42 (dd, 1H, *J* = 8.0, 1.5 Hz, ArH), 7.63–7.65 (m, 2H, AA′BB′, 2 × ArH), 7.76 ppm (dd, 1H, *J* = 8.0, 1.5 Hz, ArH); ^13^C NMR (125 MHz, CD_3_OD) *δ* = 30.4 (CH_2_), 32.5 (CH_2_), 37.8 (CH_2_), 38.2 (CH_2_), 38.9 (CH_2_), 96.5 (C), 127.9 (CH), 128.7 (CH), 129.1 (CH), 130.1 (CH), 130.2 (CH), 139.4 (C), 139.6 (C), 140.9 (CH), 148.2 (C), 165.8 (C), 174.6 ppm (C); LRMS (LC–MS ES+): *m*/*z* 597 (M+H^+^, 100%). HRMS (ES^+^): calcd for C_21_H_26_I_1_N_8_O_3_S_1_ [M+H]^+^ 597.0888, found 597.0890 (−0.29 ppm).

##### 3.2.2.18 *N*-(3-((4,6-Diamino-1,3,5-triazin-2-yl)amino)propyl)-3-(4-((2-(1-hydroxy-4-oxocyclohexa-2,5-dien-1-yl)-1*H*-indol-1-yl)sulfonyl)phenyl)propanamide (**46**)

Copper (I) iodide (0.007 mmol, 1.3 mg) and tetrakis(triphenylphosphine) palladium(0) (0.0042 mmol, 5 mg) were added to a solution of aryl iodide **47** (0.07 mmol, 42 mg) and alkyne **1** (0.0812 mmol, 11 mg) in degassed DMF (332 μL), *^i^*Pr_2_NH (140 μL) and water (28 μL) and the resultant mixture heated under microwave irradiation at 100 °C for 10 min. After cooling the reaction mixture was directly purified by reverse phase HPLC to give the product as a white solid (17 mg, 40%). ^1^H NMR (500 MHz, CD_3_OD) *δ* = 1.56 (tt, 2H, *J* = 7.0, 7.0 Hz, CH_2_), 2.45 (t, 2H, *J* = 7.5 Hz, CH_2_), 2.94 (t, 2H, *J* = 7.5 Hz, CH_2_), 3.11 (t, 2H, *J* = 7.0 Hz, CH_2_), 3.15 (t, 2H, *J* = 7.0 Hz, CH_2_), 6.25–6.29 (m, 2H, AA′BB′, 2 × CH), 6.93 (d, 1H, *J* = 0.5 Hz, ArH), 7.22–7.25 (m, 1H, ArH), 7.31–7.36 (m, 3H, 3 × ArH), 7.49–7.51 (m, 1H, ArH), 7.55–7.58 (m, 2H, AA′BB′, 2 × ArH), 7.83–7.86 (m, 2H, AA′BB′, 2 × ArH), 8.19 ppm (dd, 1H, *J* = 8.5, 1.0 Hz, ArH); ^13^C NMR (125 MHz, CD_3_OD) *δ* = 30.4 (CH_2_), 32.5 (CH_2_), 37.7 (CH_2_), 37.9 (CH_2_), 38.6 (CH_2_), 68.7 (C), 114.2 (CH), 116.6 (CH), 122.6 (CH), 125.2 (CH), 126.7 (CH), 127.8 (CH), 128.4 (CH), 130.2 (CH), 137.9 (C), 140.1 (C), 142.2 (C), 149.3 (C), 151.3 (CH), 174.4 (C), 187.4 ppm (C). Note, two quaternary ^13^C resonances are not visible. MS (LC–MS ES+): *m*/*z* 603 (M+H^+^, 100%). HRMS (ES^+^): calcd for C_29_H_31_N_8_O_5_S_1_ [M+H]^+^ 603.2133, found 603.2138 (−0.84 ppm).
