# Supplementary material for: Quinol derivatives as potential trypanocidal agents
Source: Bioorg Med Chem. 2012 Feb 15;20(4):1607–15. doi: 10.1016/j.bmc.2011.12.018 (PMC3281193; doi:10.1016/j.bmc.2011.12.018)
Supplement: Supplementary data [file mmc1.pdf]

## Supporting information for:

# Quinol Derivatives as Potential Trypanocidal Agents.

Amy Capes,[¶][a] Stephen Patterson,[¶][a] Susan Wyllie,[a] Irene Hallyburton,[a] Iain T. Collie,[a] Andrew J. McCarroll,[b] Malcolm F. G. Stevens,[b] Julie A. Frearson,[a] Paul G. Wyatt,[a] Alan H. Fairlamb,[a] Ian H. Gilbert*[a]

## Contents

Compound **3** $^{1}$H-NMR spectrum 2

Compound **3** $^{13}$C-NMR spectrum 3

Compound **12** $^{1}$H-NMR spectrum 4

Compound **12** $^{13}$C-NMR spectrum 5

Compound **19** $^{1}$H-NMR spectrum 6

Compound **19** DEPTq NMR spectrum 7

Compound **35** $^{1}$H-NMR spectrum 8

Compound **38** $^{1}$H-NMR spectrum 9

Compound **38** $^{13}$C-NMR spectrum 10

Compound **42** $^{1}$H-NMR spectrum 11

Compound **42** $^{13}$C-NMR spectrum 12

Compound **44** $^{1}$H-NMR spectrum 13

Compound **44** $^{13}$C-NMR spectrum 14

Compound **46** $^{1}$H-NMR spectrum 15

Compound **46** $^{13}$C-NMR spectrum 16

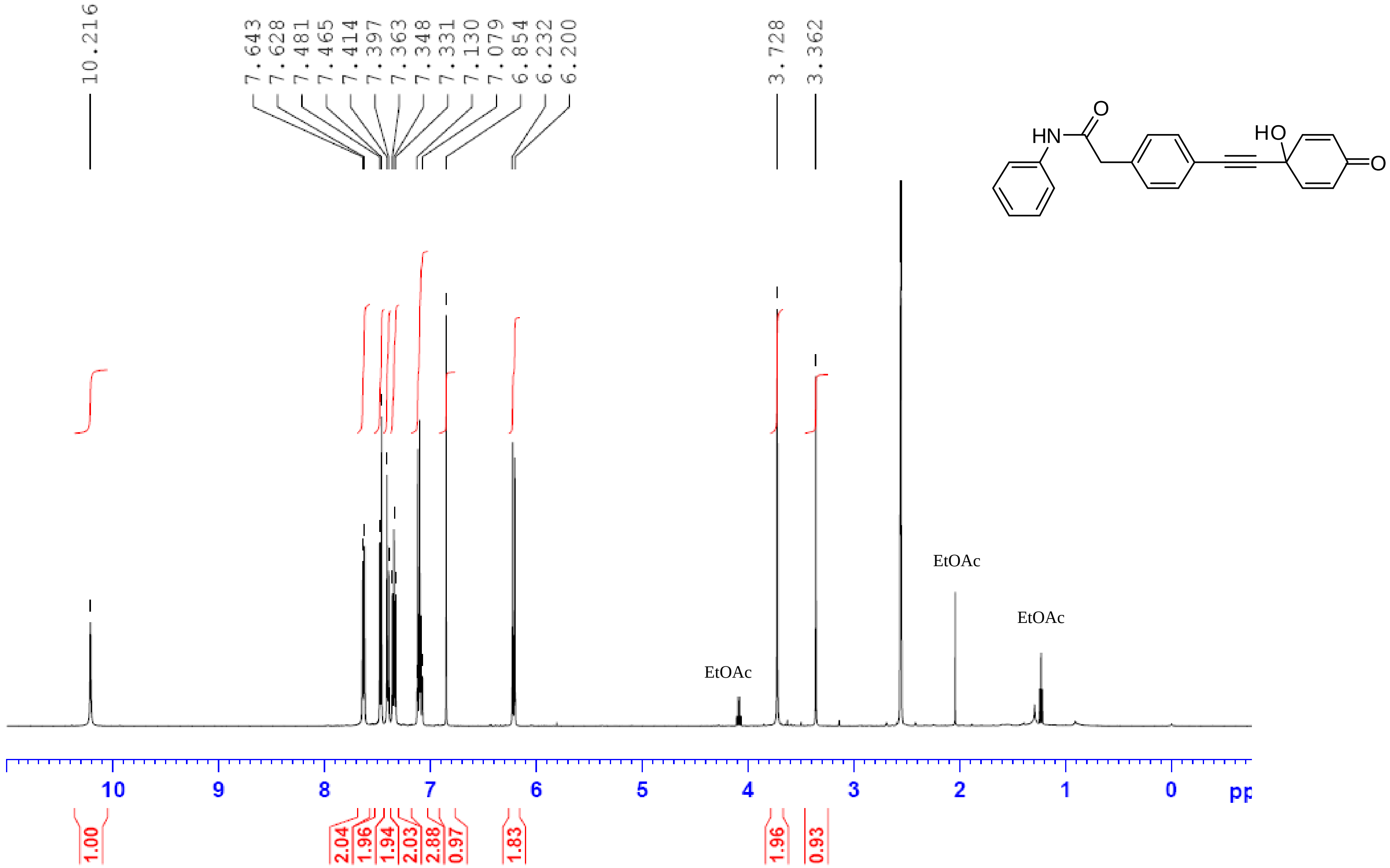


$^1$H NMR of 2-(4-((1-hydroxy-4-oxocyclohexa-2,5-dien-1-yl)ethynyl)phenyl)-*N*-phenylacetamide (compound **3**).

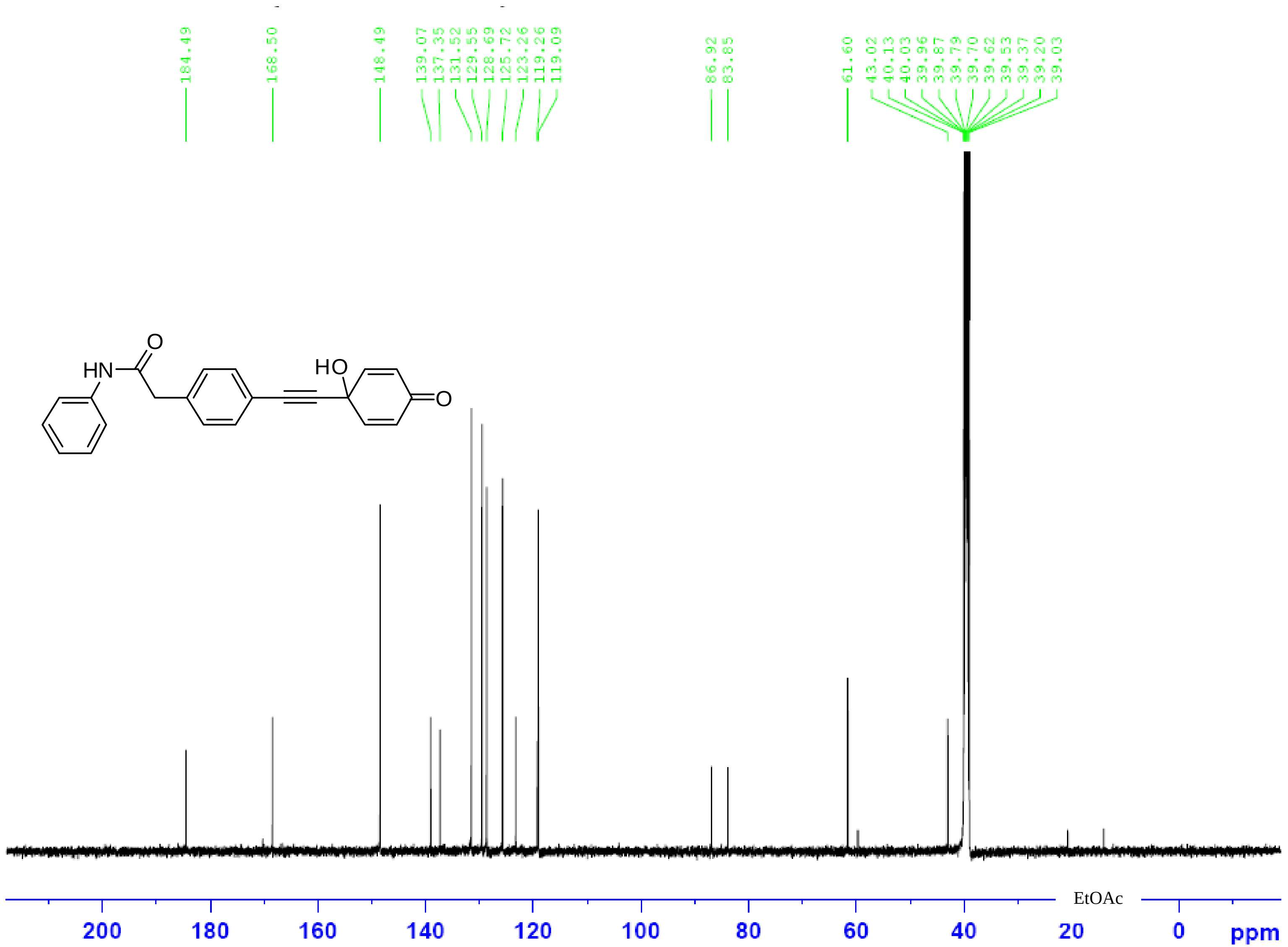


$^{13}C$ NMR of 2-(4-((1-[illegible]oxy-4-oxocyclohexa-2,5-dien-1-yl)ethynyl)ph[illegible]-N-phenylac[illegible]e (compound **3**).

EtOAc EtOAc EtOAc

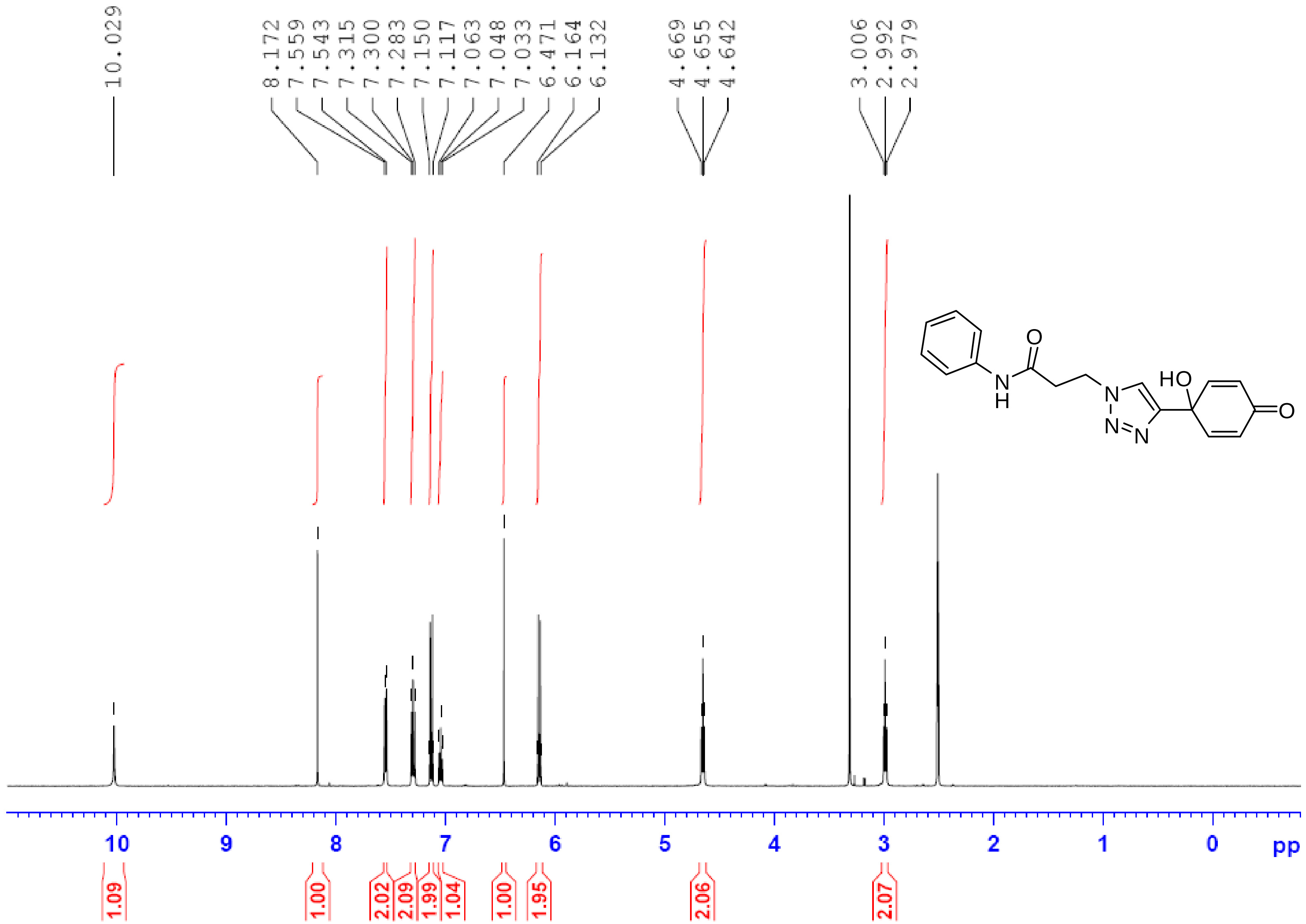


$^{1}$H NMR of 3-(4-(1-hydroxy-4-oxocyclohexa-2,5-dien-1-yl)-1*H*-1,2,3-triazol-1-yl)-*N*-phenylpropanamide (compound **12**).

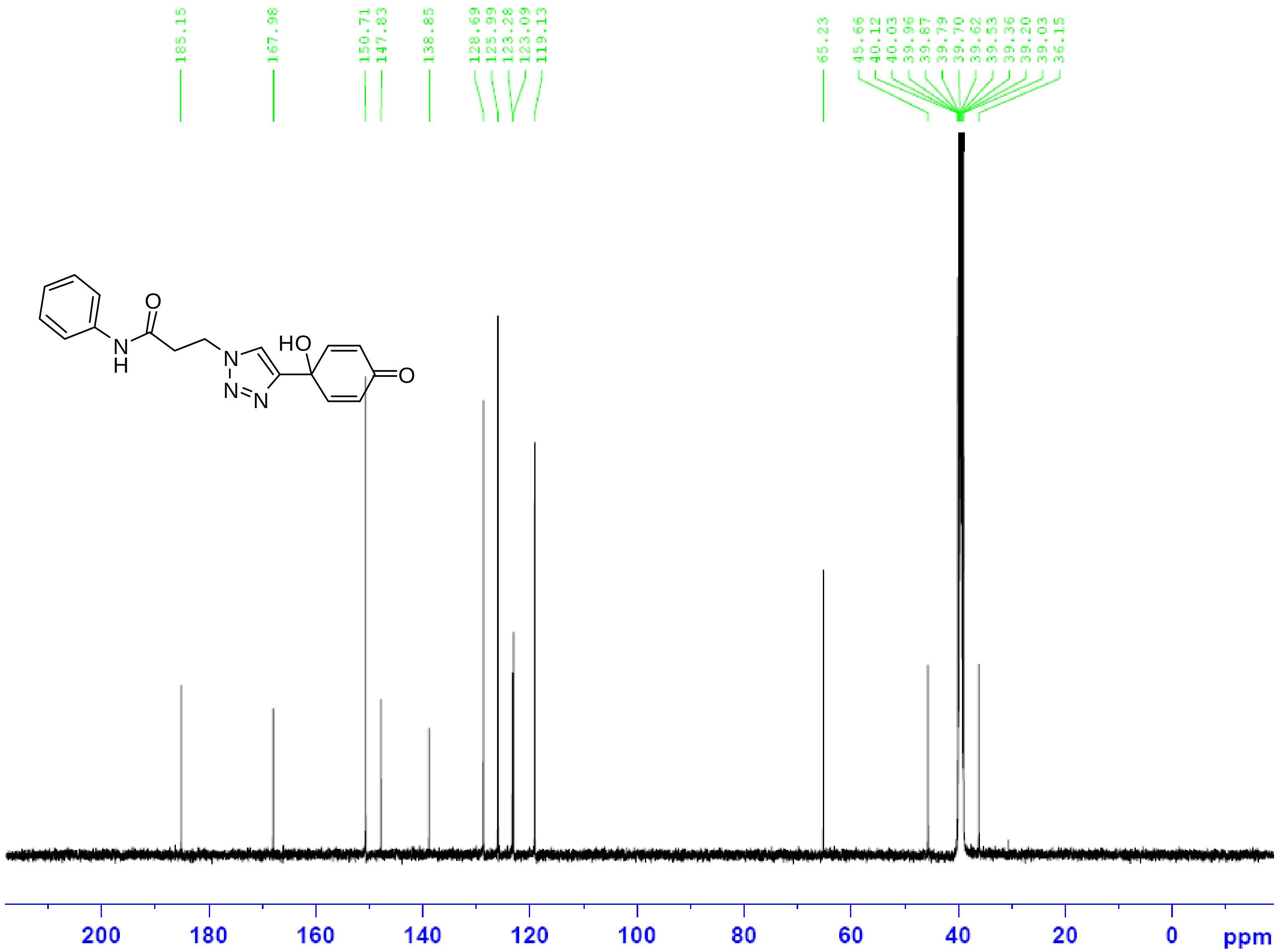


$^{13}C$ NMR of 3-(4-(1-hydroxy-4-oxocyclohexa-2,5-dien-1-yl)-1*H*-1,2,3-triazol-1-yl)-*N*-phenylpropanamide (compound **12**).

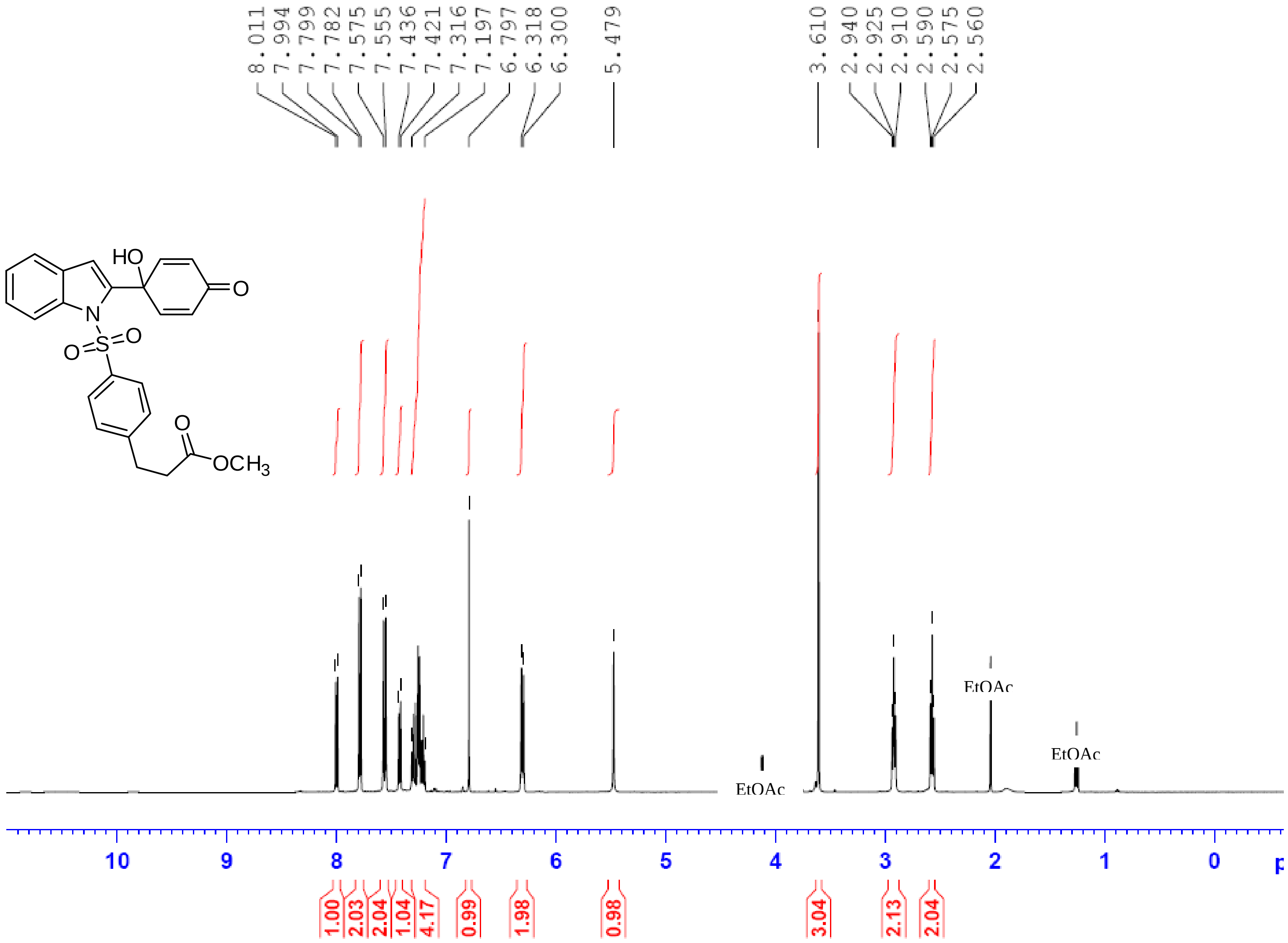


$^{1}$H-NMR of methyl 3-(4-((2-(1-hydroxy-4-oxocyclohexa-2,5-dien-1-yl)-1*H*-indol-1-yl)sulfonyl)phenyl)propanoate (compound **19**).

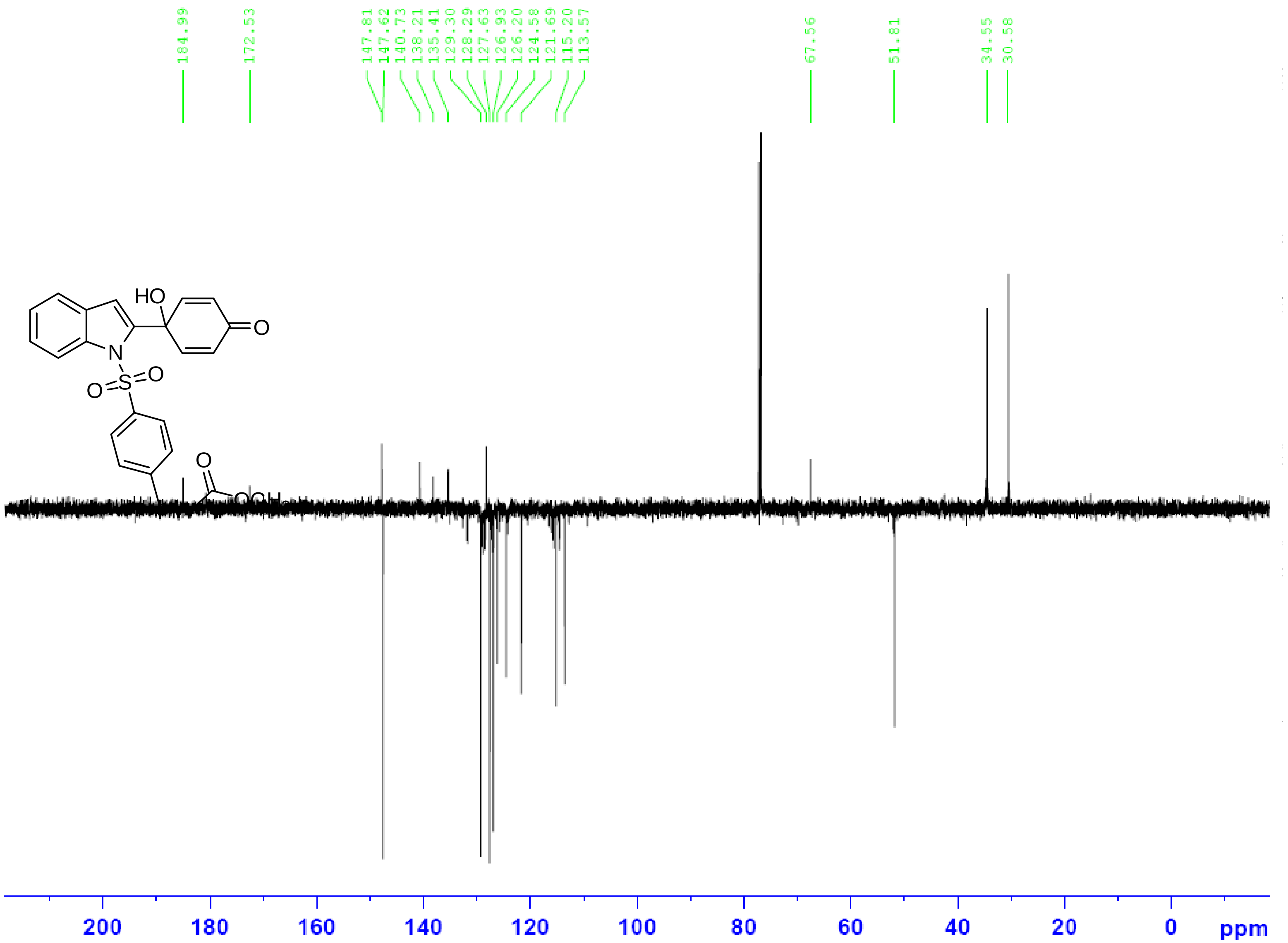


DEPTq NMR of methyl 3-(4-((2-(1-hydroxy-4-oxocyclohexa-2,5-dien-1-yl)-1*H*-indol-1-yl)sulfonyl)phenyl)propanoate (compound **19**).

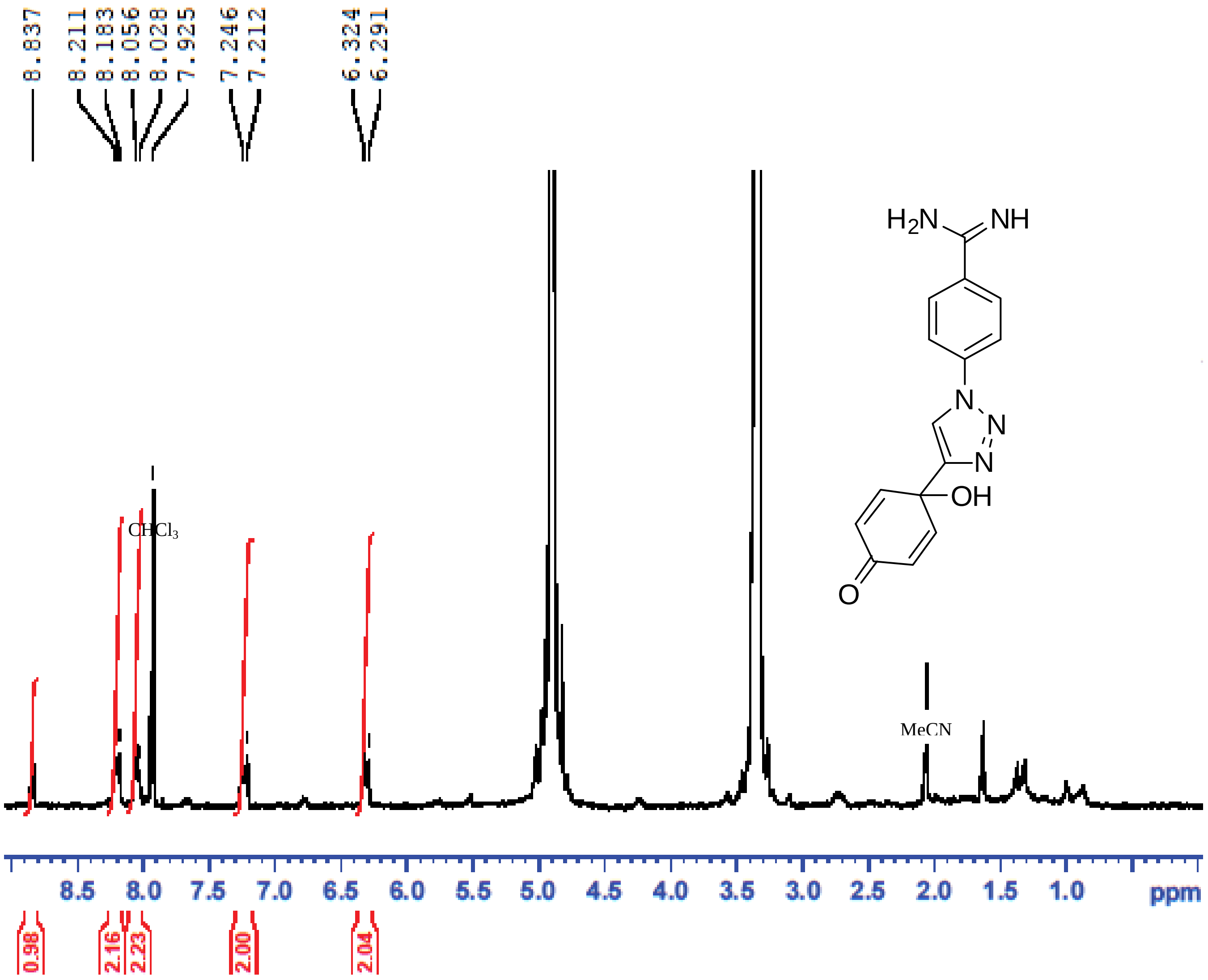


$^{1}$H-NMR of 4-(4-(1-hydroxy-4-oxocyclohexa-2,5-dienyl)-1H-1,2,3-triazol-1-yl)benzimidamide (compound **35**).

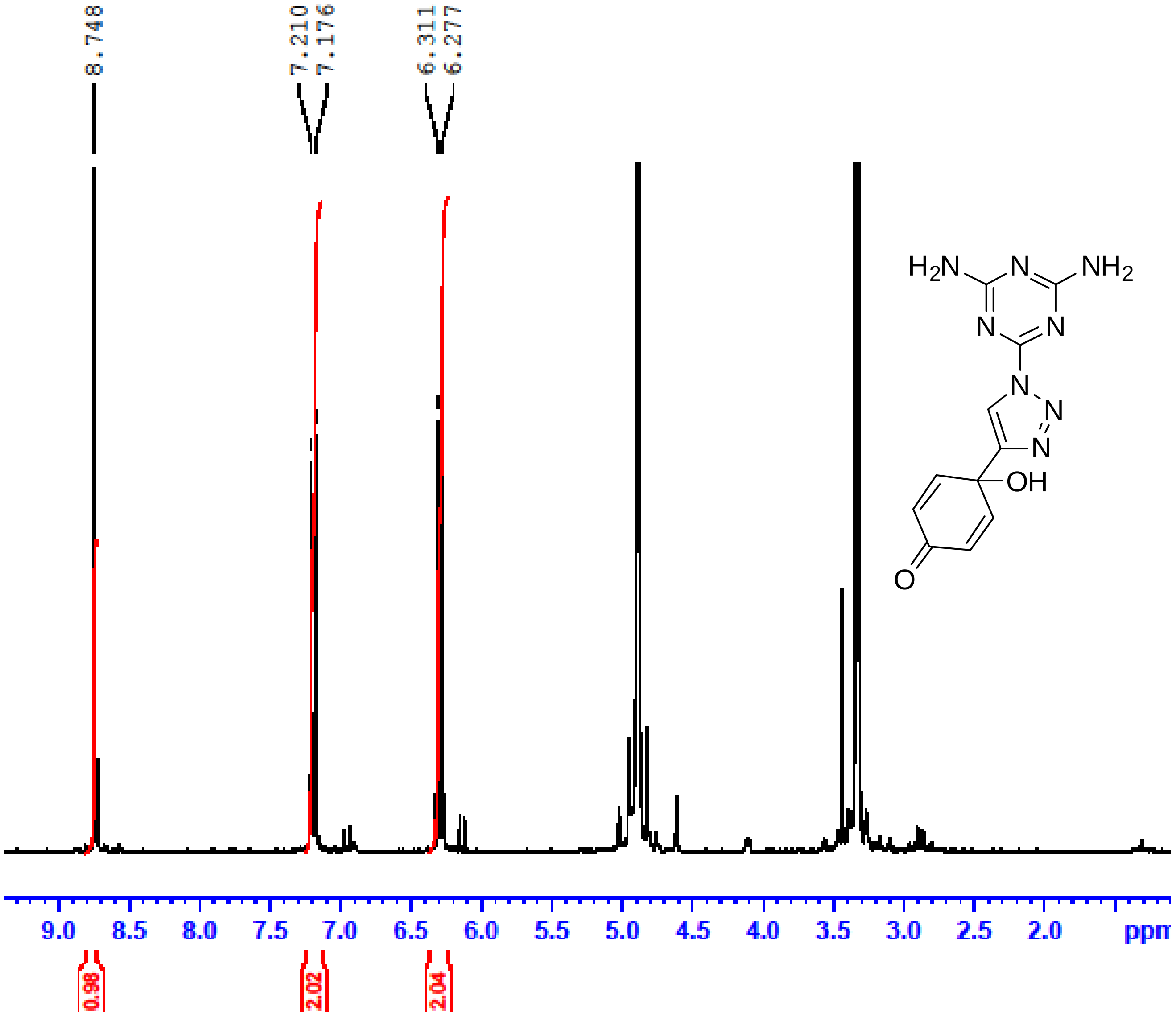


$^{1}$H-NMR of 4-(1-(4,6-diamino-1,3,5-triazin-2-yl)-1H-1,2,3-triazol-4-yl)-4-hydroxycyclohexa-2,5-dienone (compound **38**).

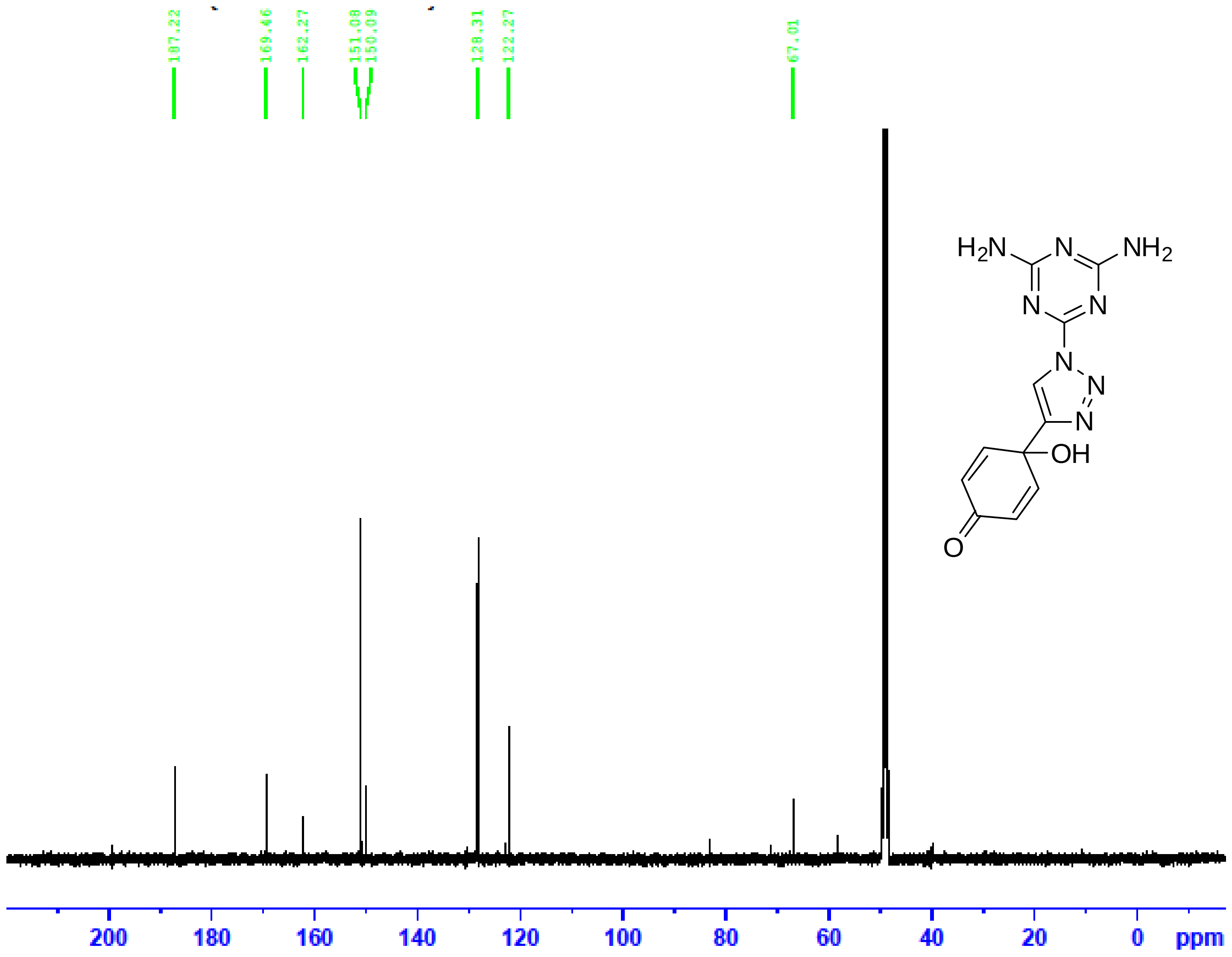


$^{13}$C-NMR of 4-(1-(4,6-diamino-1,3,5-triazin-2-yl)-1H-1,2,3-triazol-4-yl)-4-hydroxycyclohexa-2,5-dienone (compound **38**).

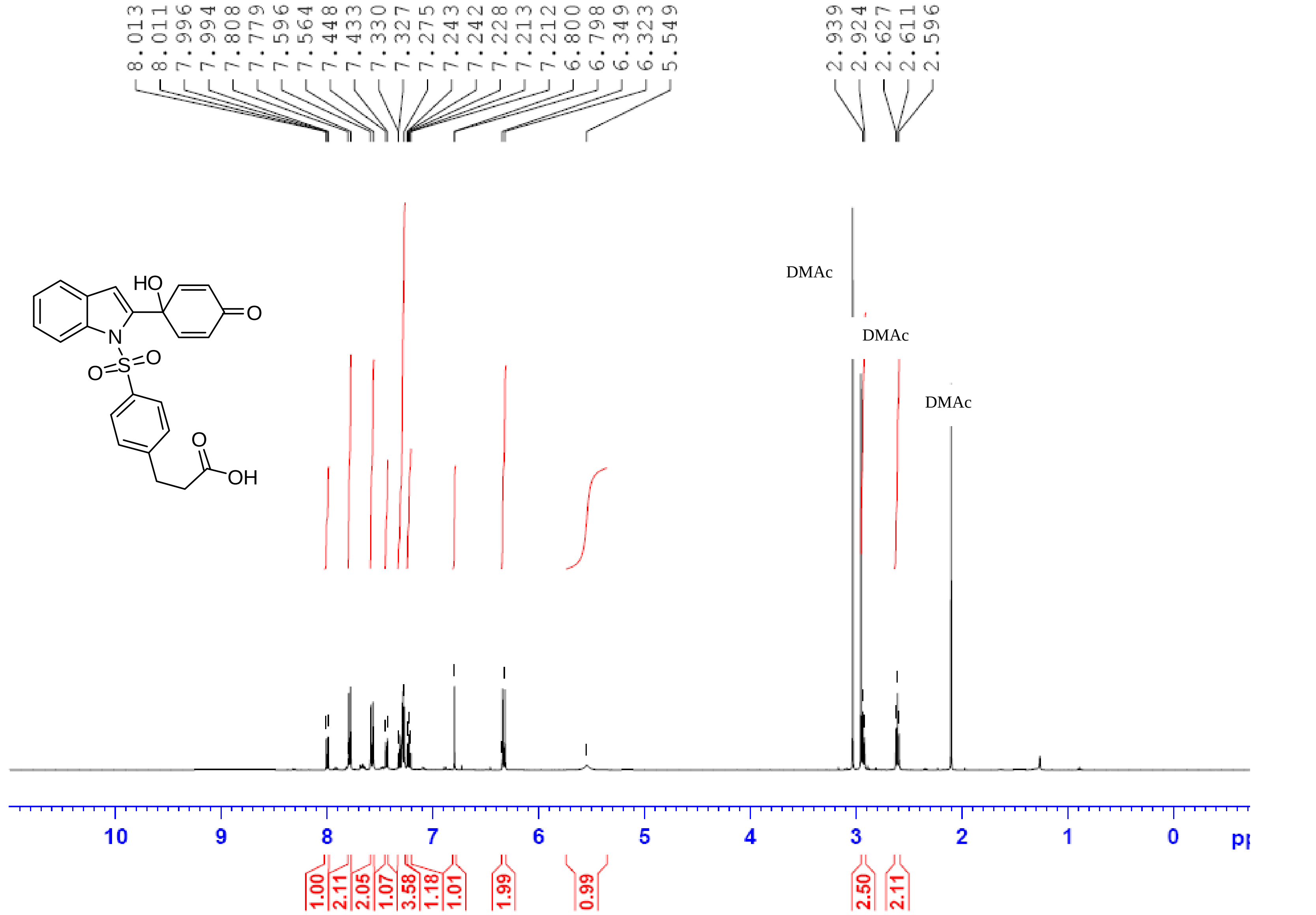


$^{1}$H-NMR of 3-(4-((2-(1-Hydroxy-4-oxocyclohexa-2,5-dien-1-yl)-1*H*-indol-1-yl)sulfonyl)phenyl)propanoic acid (compound **42**).

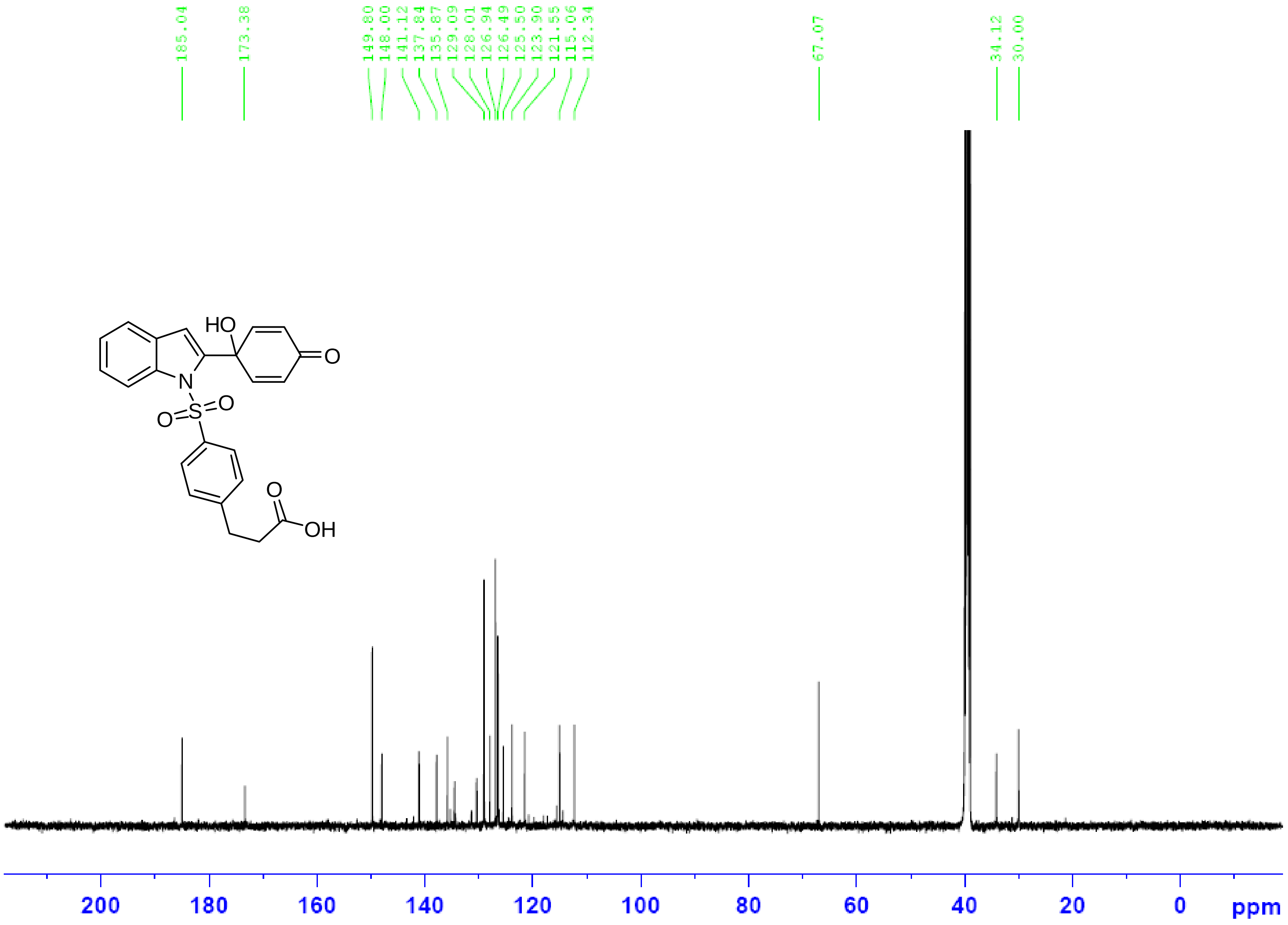


$^{13}$C-NMR of 3-(4-((2-(1-Hydroxy-4-oxocyclohexa-2,5-dien-1-yl)-1*H*-indol-1-yl)sulfonyl)phenyl)propanoic acid (compound **42**).

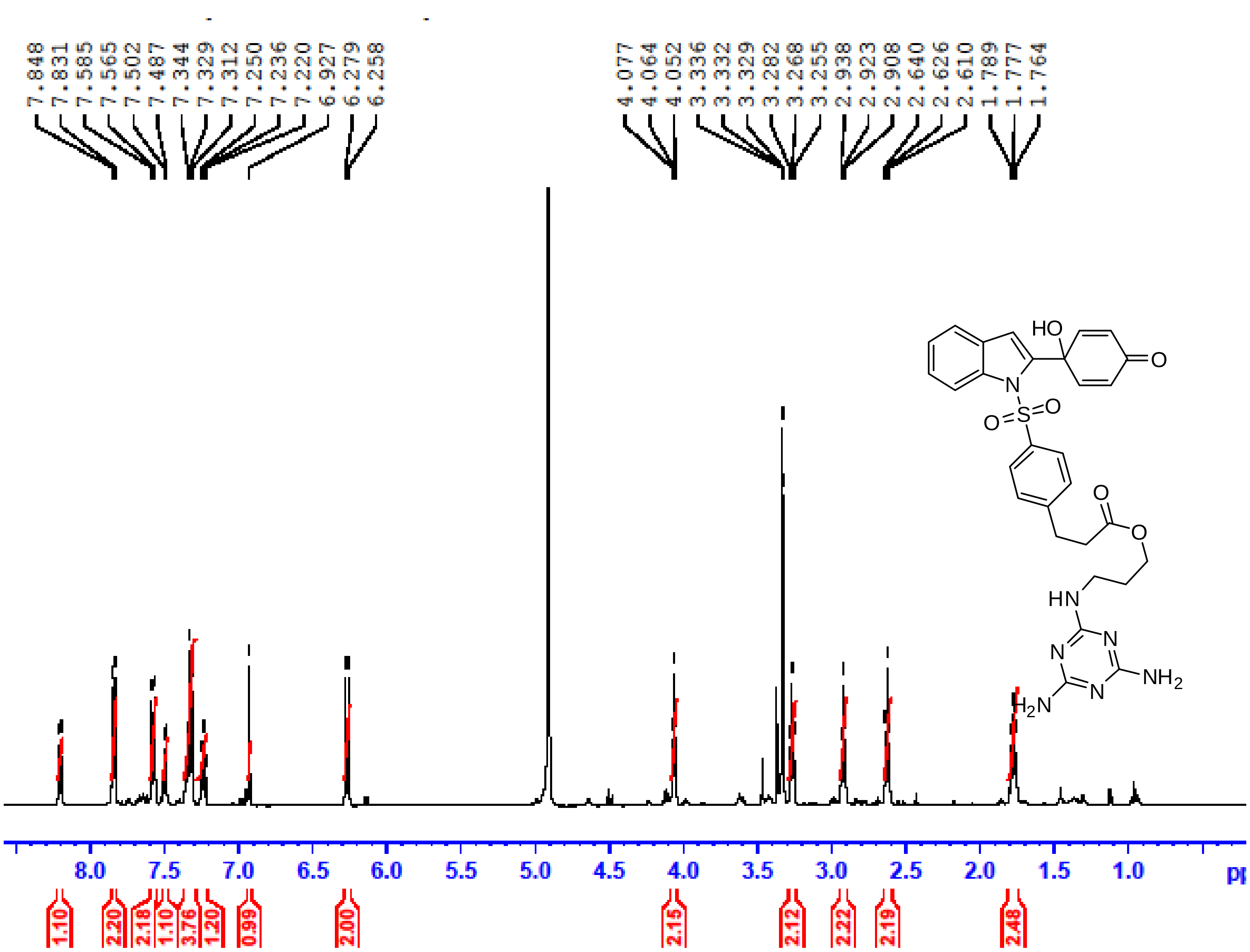


$^1$H-NMR of 3-((4,6-diamino-1,3,5-triazin-2-yl)amino)propyl 3-(4-((2-(1-hydroxy-4-oxocyclohexa-2,5-dien-1-yl) -1*H*-indol-1-yl)sulfonyl)phenyl)propanoate (compound **44**).

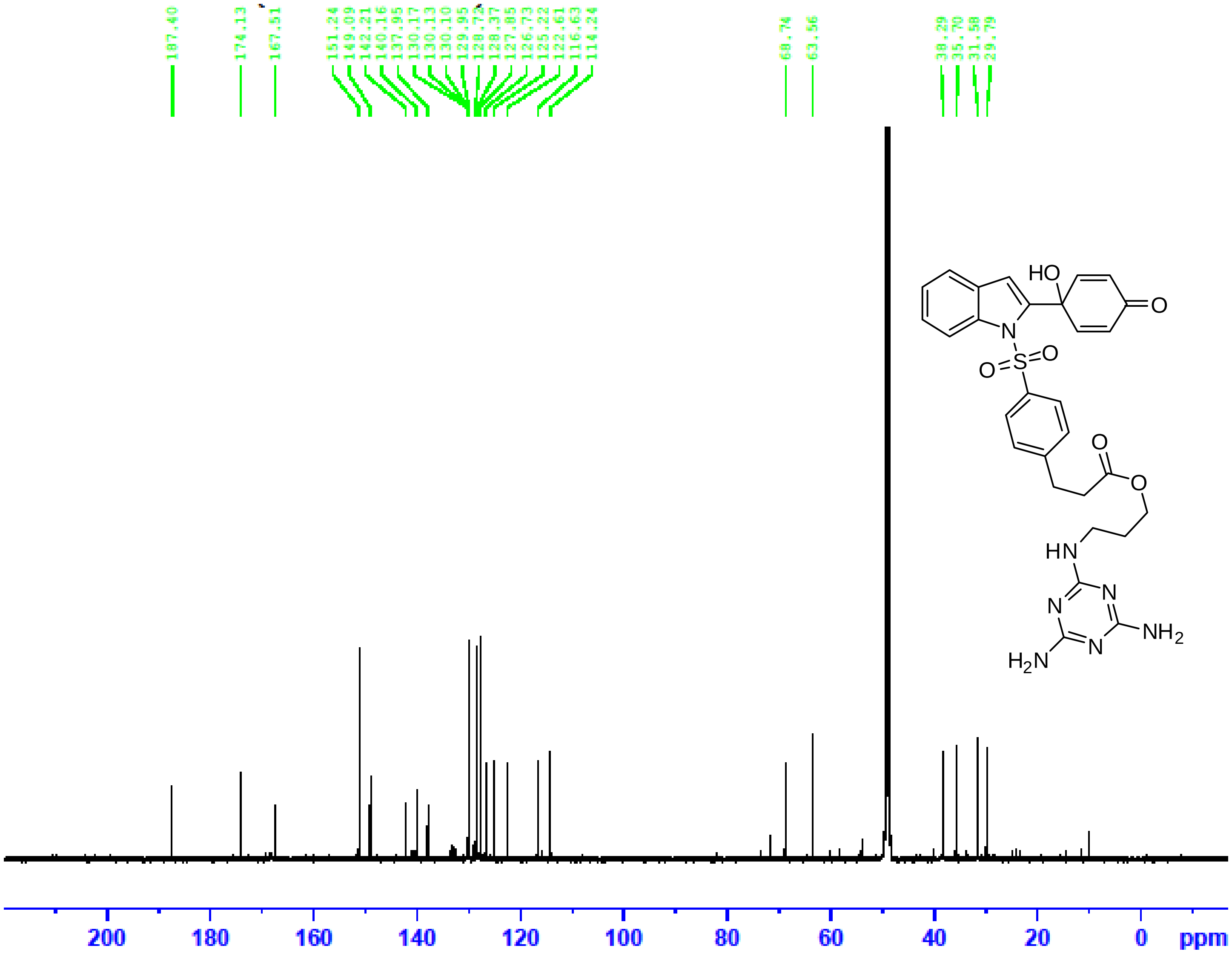


$^{13}$C-NMR of 3-((4,6-Diamino-1,3,5-triazin-2-yl)amino)propyl 3-(4-((2-(1-hydroxy-4-oxocyclohexa-2,5-dien-1-yl) -1*H*-indol-1-yl)sulfonyl)phenyl)propanoate (compound **44**).

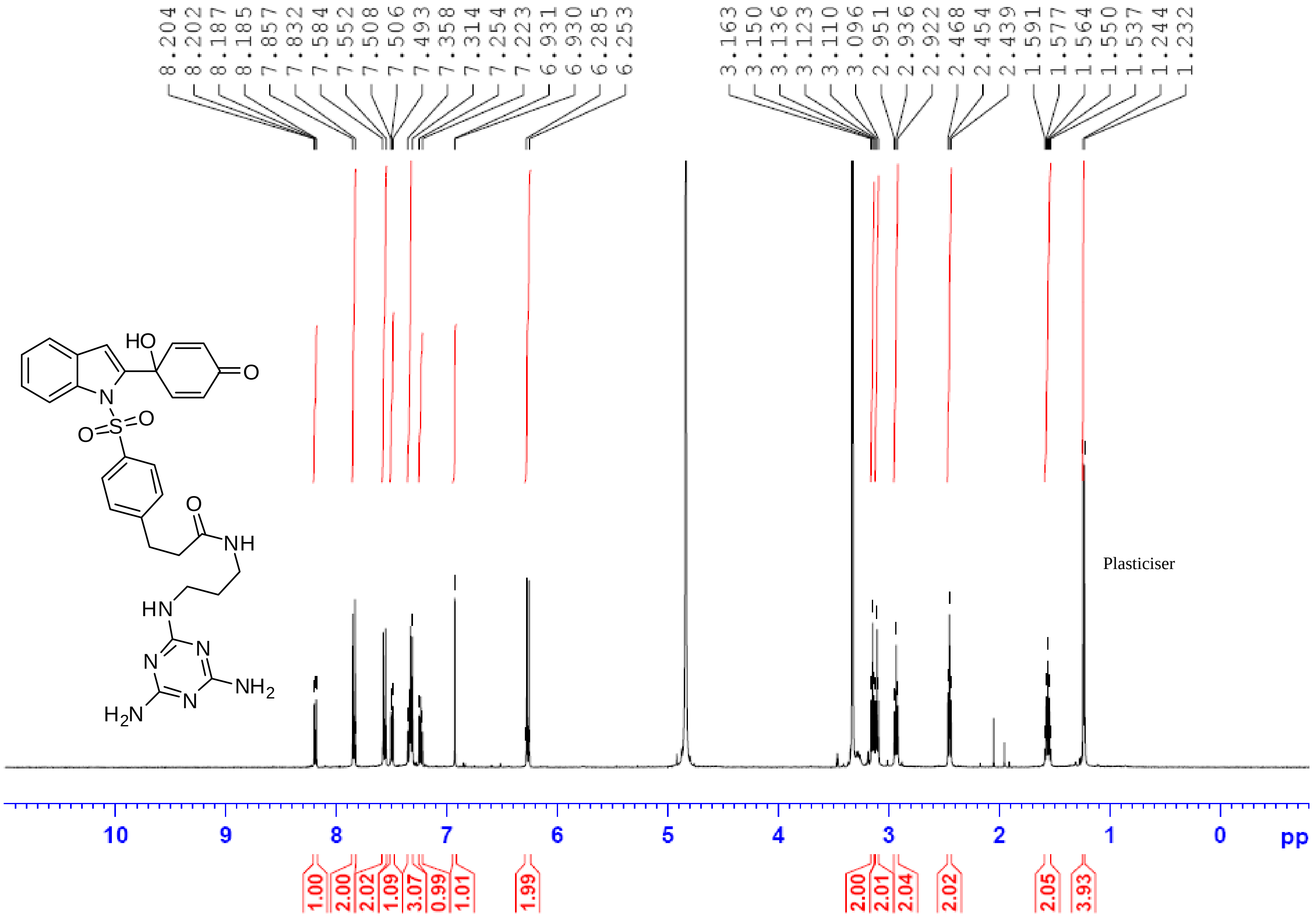


$^{1}$H-NMR of *N*-(3-((4,6-Diamino-1,3,5-triazin-2-yl)amino)propyl)-3-(4-((2-(1-hydroxy-4-oxocyclohexa-2,5-dien-1-yl)-1*H*-indol-1-yl)sulfonyl)phenyl)propanamide (compound **46**).

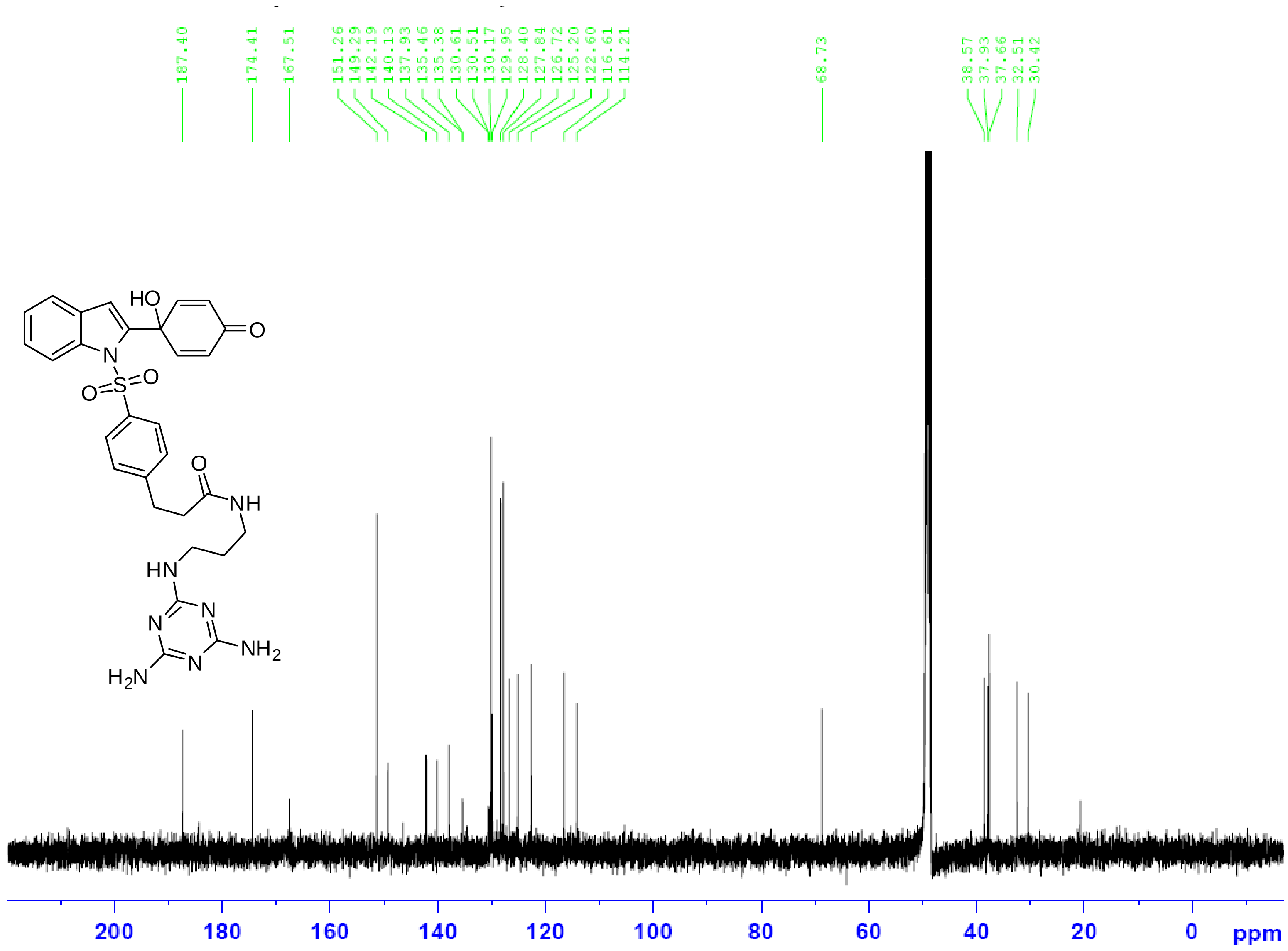


$^{13}$C-NMR of *N*-(3-((4,6-Diamino-1,3,5-triazin-2-yl)amino)propyl)-3-(4-((2-(1-hydroxy-4-oxocyclohexa-2,5-dien-1-yl)-1*H*-indol-1-yl)sulfonyl)phenyl)propanamide (compound **46**).
